# A divergent route to core- and peripherally functionalized diazacoronenes that act as colorimetric and fluorescence proton sensors†
†Electronic supplementary information (ESI) available: Experimental details, synthesis and characterization of **TAC**s and **BAC**s, computational details, X-ray crystallographic information files. CCDC 1031859, 1031861 and 1032434. For ESI and crystallographic data in CIF or other electronic format see DOI: 10.1039/c5sc00304k



**DOI:** 10.1039/c5sc00304k

**Published:** 2015-03-31

**Authors:** Bo He, Jing Dai, Danylo Zherebetskyy, Teresa L. Chen, Benjamin A. Zhang, Simon J. Teat, Qichun Zhang, Linwang Wang, Yi Liu

**Affiliations:** a The Molecular Foundry , Lawrence Berkeley National Laboratory , Berkeley , California 94720 , USA . Email: yliu@lbl.gov; b Department of Chemistry , Zhejiang University , Hangzhou , 310027 , China; c Materials Sciences Division , Lawrence Berkeley National Laboratory , Berkeley , California 94720 , USA; d Advanced Light Source , Lawrence Berkeley National Laboratory , Berkeley , California 94720 , USA; e School of Materials Science and Engineering , Nanyang Technological University , 50 Nanyang Avenue , 639798 , Singapore

## Abstract

Combining core annulation and peripheral group modification, we have demonstrated a divergent synthesis of a family of highly functionalized coronene derivatives from a readily accessible dichlorodiazaperylene intermediate. Various reactions, such as aromatic nucleophilic substitution, Kumada coupling and Suzuki coupling proceed effectively on α-positions of the pyridine sites, giving rise to alkoxy, thioalkyl, alkyl or aryl substituted polycyclic aromatic hydrocarbons. In addition to peripheral group modulation, the aromatic core structures can be altered by annulation with thiophene or benzene ring systems. Corresponding single crystal X-ray diffraction and optical studies indicate that the heteroatom linkages not only impact the solid state packing, but also significantly influence the optoelectronic properties. Moreover, these azacoronene derivatives display significant acid-induced spectroscopic changes, suggesting their great potential as colorimetric and fluorescence proton sensors.

## Abstract 2

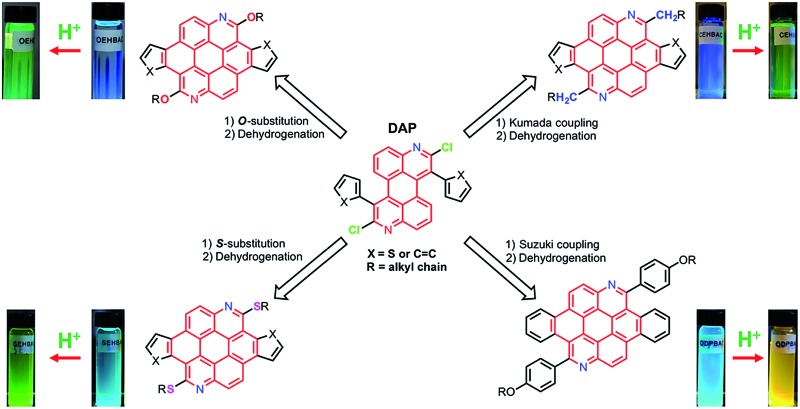
One-stop center for functional polycyclic aromatic hydrocarbons — a dichlorodiazaperylene intermediate has been synthesized and employed for the synthesis of highly functionalized coronene derivatives.

## Introduction

Polycyclic aromatic hydrocarbons (PAHs) and heterocyclic PAHs containing multiple benzenoid aromatic units, which can be considered as a small segment of graphene sheet,^1^ have drawn much attention for potential applications in supramolecular electronics.^2^ The large conjugated π-surfaces facilitate delocalization of electrons while the rigid flat geometry is conducive for strong π-stacking interactions. The former feature affects tuning of frontier molecular orbital energies and the related optoelectronic properties, while the latter is essential for intermolecular charge transport *via* a hopping mechanism.^3^ The combined structural features render PAHs excellent candidates for organic semiconductors. Structural tuning of these molecular systems can be realized either on the aromatic core, typically through ring annulation or by introducing different heteroatoms, or by changing the peripheral groups. Two major types of PAHs are linear acenes^4^ and radially expanded benzenoids such as coronenes and annulated coroenes.^5^ Different heteroatoms, such as B,^6^ N,^6,7^ O,^8^ P,^9^ and S,^10^ have also been incorporated to the core. On the other hand, the peripheral groups are known to affect the solubility, the intermolecular packing in the solid states, and the electronic structures, all relating to the anchoring atom between the aromatic core and the solubilizing alkyl chains. Commonly used peripheral substituents for PAHs are alkyl and alkoxy groups.^7*i*^ Other peripheral substituents, such as thioalkyl^11^ and aryl groups,^12^ although less studied, have been shown to affect the energy levels as well as the packing in the solid state, all of which are very relevant for tuning optoelectronic properties.

**Scheme 1:**
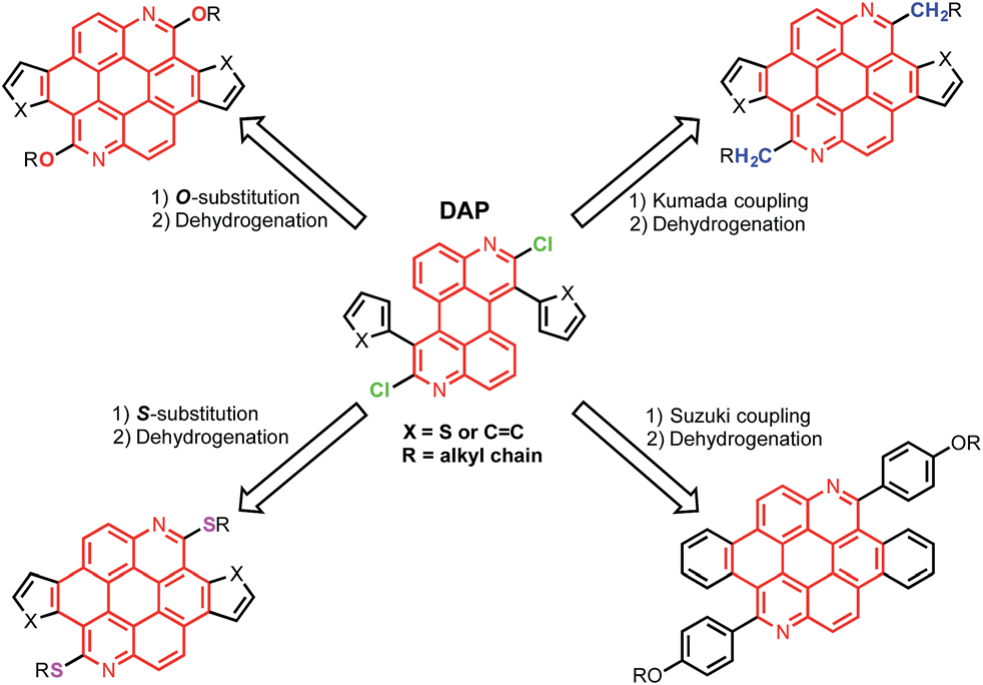
Synthetic scheme illustrating the divergent synthesis of highly functionalized azacoronenes from the intermediate **DAP**.

Thienoazacoronene (**TAC**) derivatives are one of the very few PAH monomers that have recently been employed in high performance conjugated polymers.^13^ The structure of **TAC** features (Scheme 1) two nitrogen atoms on the coronene core, two annulated thiophene units and two alkoxy peripheral groups, all together endowing the TAC-based polymers a low ionization potential and a high open circuit voltage (*V*_oc_) of 0.89 V in organic solar cells. Considering that the electron donating alkoxy group often leads to a reduction of the ionization potential, it is imperative to explore structural tuning of this multi-functional aromatic core by changing the peripheral groups. Herein we report a divergent synthetic route that involves a versatile and readily accessible intermediate, 1,7-diaryl-2,8-dichloro-3,9-diazaperylene (**DAP**). The 2,8-dichloro-substitution allows the introduction of peripheral groups with different heteroatom linkage *via* various routes, such as nucleophilic aromatic substitution (S_N_Ar), Kumada coupling and Suzuki coupling reactions. The scope is further expanded when different aryl groups are introduced to the 1,7-position of the azaperylene, leading to diverse aromatic systems after simple transformations. The combined modifications of peripheral group and core structure have a great impact on properties of the PAHs, such as the solution optical properties and the solid state packing, as characterized by UV-vis/photoluminescence spectroscopies and single crystal X-ray analysis. Moreover, the PAHs form adducts with acids due to the presence of nitrogen heteroatoms in the azacoronenes. Significant colour and fluorescence changes are observed upon mixing the azacoronenes with acids, suggesting their great potential as dual-mode pH sensors.

## Results and discussion

### Synthesis of azacoronenes

**Scheme 2:**
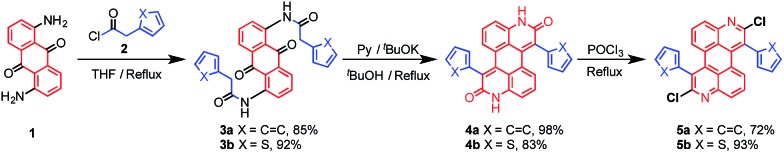
Synthesis of substituted **DAP**s.

The syntheses of dichloroazaperylenes (**DAP**s) are depicted in Scheme 2. The reaction between the commercially available 1,5-diaminoanthraquinone (**1**) and 2-arylacetyl chloride gave the diamides **3a** and **3b**, which underwent double intramolecular Knoevenagel condensation to afford the fused diamides **4a** and **4b**. The subsequent treatment with POCl_3_ gave the aromatized **DAP5a** and **5b**. Aryl substituents, such as phenyl and thienyl groups were readily introduced to the bay positions of **DAP**s *via* this reaction sequence.

**Scheme 3:**
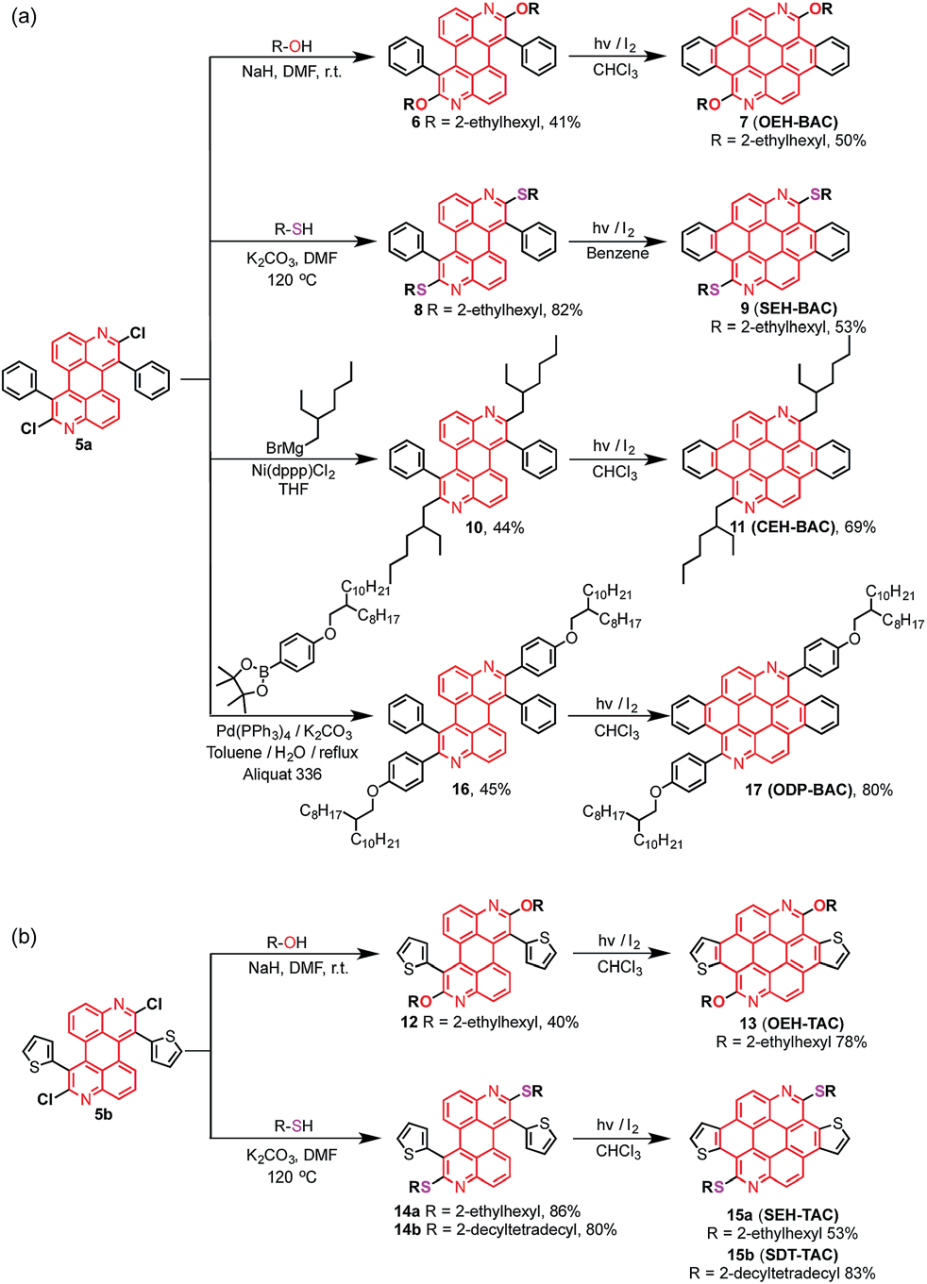
Various reactions of (a) phenyl-**DAP** and (b) thienyl-**DAP** for the synthesis of substituted **BAC**s and **TAC**s.

The chlorides in **DAP**s are activated by the adjacent nitrogen atoms, which can undergo nucleophilic aromatic substitution and metal-catalyzed coupling reactions. As shown in Scheme 3a, substitution of the chlorides on **5** is very effective when alcohols and thiols are used in the presence of a suitable base to give the corresponding PAHs with alkoxy and thioalkyl peripheral substituents. The reactions between **5a** and alkyl thiols went smoothly when potassium carbonate was employed as the base. In the case of alcohols, a stronger base, such as NaH, was required to furnish the reaction. Further oxidative dehydrogenation ring closure went smoothly to give the previously unknown benzoazacoronene (**BAC**) series when the precursors solutions were irradiated under UV light in the presence of catalytic amount of I_2_. Similar reaction sequences were applied to the thiophene derived **DAP5b** to give the thienoazacoronene (**TAC**) series (Scheme 3b). It is noteworthy that the S_N_Ar reaction can greatly facilitate the investigation of side chain effects, since different alkoxy and thioalkyl chains can be readily introduced. As demonstrated in Scheme 3b, the 2-ethylhexyl (EH) and 2-decyltetradecyl (DT) chains are attached to the **TAC** core through sulfur linkage following this protocol.

In addition to nucleophilic substitutions, various metal-catalyzed coupling reactions work effectively on the **DAP**s to give alkyl or aryl substituted PAHs. As shown in Scheme 3a, the coupling between alkyl magnesium bromide and **DAP5a** went smoothly in the presence of Ni(ii) catalyst to yield the carbon linked **CEH-BAC** (**11**). Under Suzuki coupling reaction conditions, aryl substituent was introduced to the 2,8-positions of the **BAC** core (Scheme 3a). Compared to the previous synthesis of **TAC**s^13^ which only yielded *O*-alkylated products as a result of the necessary alkylation–aromatization step, this **DAP**-based sequence is much more versatile.

### Single crystal X-ray structure analysis

**Fig. 1:**
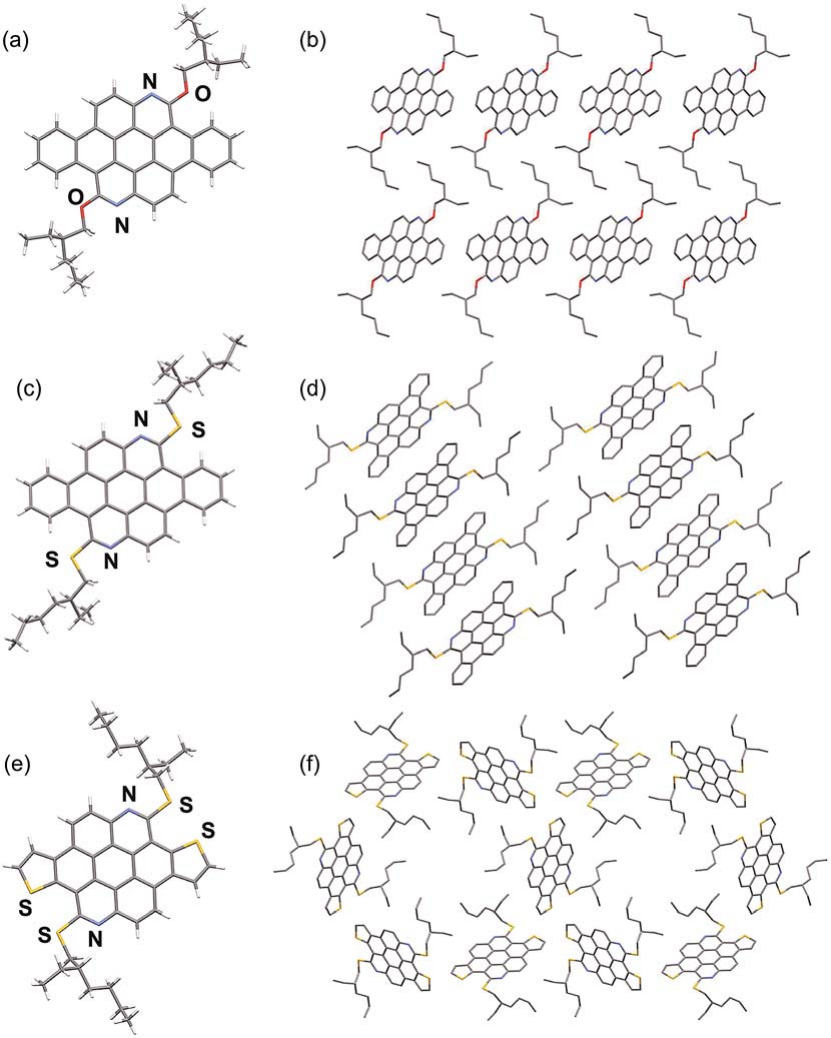
Capped stick representation of X-ray structures of **OEH-BAC** (a and b), **SEH-BAC** (c and d) and **SEH-TAC** (e and f). (a, c, e) front views; (b, d, f) top views (H atoms omitted for clarity) of the stacked structure showing intercolumnar arrangement of **OEH-BAC**, **SEH-BAC** and **SEH-TAC**, respectively.

Single crystal X-ray structure analysis of the azacoronenes provides direct information about the molecular structure and packing. The solid-state structures of **OEH-BAC** (**7**) and **SEH-BAC** (**9**) share great similarity to the reported **OEH-TAC** (**13**) analogue (Fig. 1). The aromatic **BAC** cores of **OEH-BAC** and **SEH-BAC** adopt planar conformations, which stack into 1D columnar structures along the crystallographic *b*-axis with inter-plane spacing of 3.40 and 3.32 Å, and a centroid-to-centroid distance of 5.20 Å and 5.21 Å between two adjacent **BAC** cores, respectively. Neighbouring columns are arranged in a slipped herringbone stacking geometry, with nearly half of **BAC**'s aromatic surface overlapping with the nearby ones. The single crystal X-ray structure of the thioalkyl substituted **SEH-TAC** (**15a**) reveals that the nearly flat aromatic surfaces stack into columns similar to these observed in **OEH-TAC**, with an inter-plane spacing and centroid-to-centroid distance of 3.36 Å and 5.06 Å, respectively. The intermolecular packing is however very different from **OEH-TAC**. As shown in Fig. 1e and f, hexagonally and triangularly arranged 1D-stacked columns are interconnected in the solid state of **SEH-TAC**, as opposed to the parallel arrangement of columns in **BAC**s and **OEH-TAC**.^13^ Such structural differences illustrate that the heteroatom linkage between the PAH core and the alkyl group could have a profound impact on the solid-state packing.

### UV-vis, photoluminescence (PL), and electrochemical studies

**Fig. 2:**
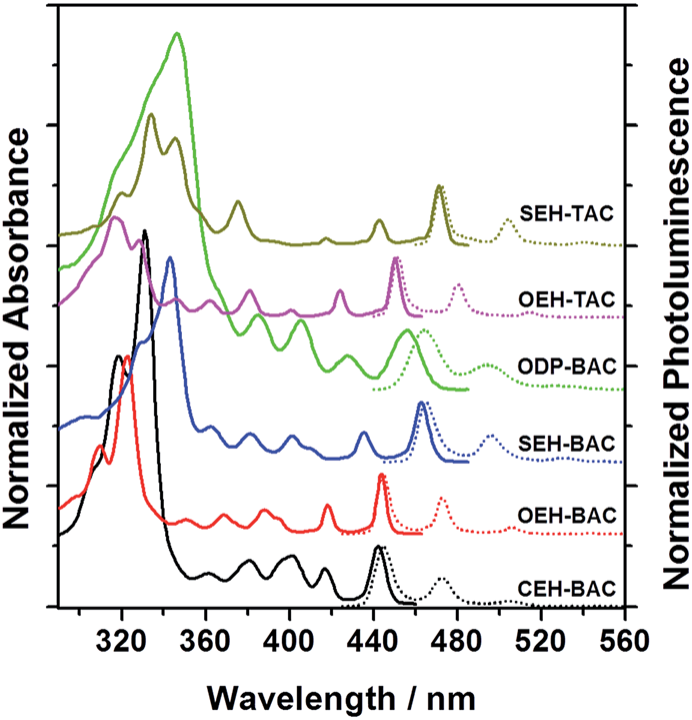
Normalized UV-vis (solid lines) and PL (dotted lines) spectra of **BAC** and **TAC** derivatives (solvent: toluene).

The different heteroatom linkages not only influence the solid-state packing, but more significantly the optical properties of the PAHs. A comparison of the UV-vis spectra (Fig. 2 and Table 1) of the **BAC** and **TAC** derivatives shows the following features: (a) well-separated vibronic peaks at low energies are present in both **TAC**s and **BAC**s; (b) the lowest energy transitions of **BAC** are blue-shifted compared to these of the **TAC**s by ∼7 nm; (c) the thioalkyl substitution shifts the transitions to the longer wavelength when compared to the alkoxy substituted ones by ∼20 nm, while the alkyl substitution shifts the transitions to the shorter wavelength; in addition, the absorption of the phenyl-substituted **BAC** is in between the thioalkyl and alkoxy substituted ones and exhibits broadened peaks, suggestive of extended conjugation and increased number of vibrations upon the attachment of the phenyl substituents.^14^ Cyclic voltammetric measurements revealed electrochemically irreversible oxidation and reduction peaks for all the azacoronene derivatives (Fig. S1, ESI†). All the HOMO and LUMO energy levels are estimated to be around –5.3–5.4 eV and –2.5–2.7 eV, respectively (Fig. S1†). It is noteworthy that the relative order should be dealt with care since the differences are within the error of the method, which are further obscured by electrochemical irreversibility.

**Table 1:** List of absorption and emission properties^*a*^

Compd	*λ*_abs_ ^*b*^ (nm)	*λ*_PL_ ^*c*^ (nm)	*E* opt g ^*d*^ (eV)	QY^*e*^ (*Φ*)
**OEH-BAC**	444	445	2.75	0.92
**OEH-BAC-H** ^*f*^	467	476	2.15	0.70
**SEH-BAC**	463	465	2.62	0.24
**SEH-BAC-H** ^*f*^	506	517	1.97	0.08
**CEH-BAC**	442	445	2.75	0.50
**CEH-BAC-H** ^*f*^	474	484	2.09	0.19
**OEH-TAC**	450	452	2.71	0.17
**OEH-TAC-H** ^*f*^	480	491	2.04	0.26
**SEH-TAC**	471	472	2.59	0.11
**SEH-TAC-H** ^*f*^	514	540	1.99	0.05
**ODP-BAC**	456	464	2.64	0.36
**ODP-BAC-H** ^*f*^	484	523	2.41	0.41

^*a*^In toluene.

^*b*^Absorption at the longest wavelength.

^*c*^Emission at the shortest wavelength, excited at 365 nm.

^*d*^Optical bandgap, determined by 1240/*λ*onsetabs.

^*e*^DPA (9,10-diphenylanthrancene) was used as the standard (in toluene, *Φ*^DPA^ = 0.90).

^*f*^Acid treated azacoronenes.

The emission spectra of all the annulated azacoronenes display pronounced effective vibronic progressions that are dominated by three peaks, with the highest energy peak being the most intense one (Fig. 2). Another noticeable feature is the very small Stokes shift between the highest energy peak in the PL spectrum and the lowest energy peak in the absorption, typically within a few nanometers except for that of **ODP-BAC**, which is presumably associated with non-planarity induced by the aryl groups. A comparison of the respective quantum yields (QY) indicates (Table 1) that the incorporation of S atoms, either on the periphery or the core, lowers the quantum yields significantly. For example, the QY decreases from 0.92 to 0.24 when changing from **OEH-BAC** to **SEH-BAC**. The **TAC**s are much less fluorescent when compared to the **BAC** counterparts, with QYs of 0.17 and 0.11 for **OEH-TAC** and **SEH-TAC**, respectively. The **BAC**s with C-linked substituents, **CEH-BAC** and **ODP-BAC**, have intermediate quantum yields of 0.50 and 0.36, respectively. The thin films of the azacoronene derivatives are highly fluorescent (Fig. S2†), suggesting that these compounds could be used for solid-state light emitting devices.

### Acid-induced spectroscopic changes

**Fig. 3:**
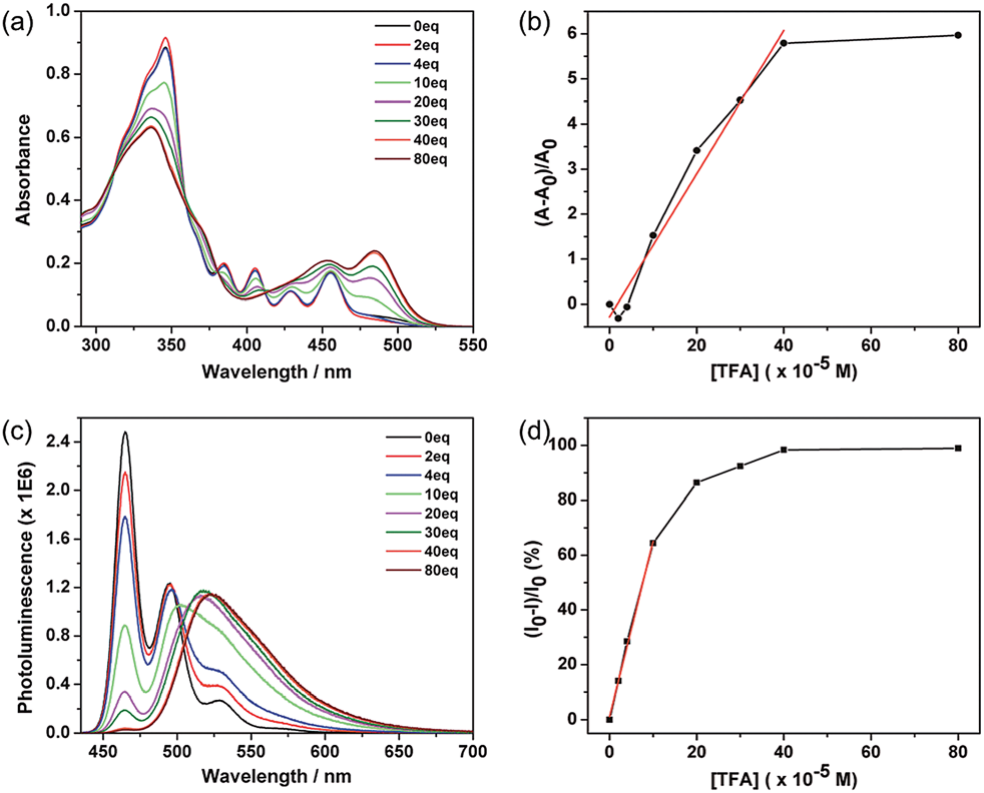
(a) UV-vis absorbance and (b) the relative increase in absorption (measured at 484 nm) of a toluene solution of **ODP-BAC** (10 μM) as a function of the concentration of TFA added; the data points in low concentration range (0–400 μM) are fitted by a linear relationship (*R*^2^ = 0.975); (c) photoluminescence and (d) the relative decrease in emission intensity (measured at 465 nm) of the same solution shown in (a) as a function of the concentration of TFA added; the data points in low concentration range (0–100 μM) are fitted by a linear relationship (*R*^2^ = 0.987).

These azacoronene derivatives show very notable spectroscopic changes when mixed with acid, as exemplified in the acid titration experiment of **ODP-BAC**. A new peak at 484 nm grew in as various amount of trifluoroacetic acid (TFA) was added into the toluene solution of **ODP-BAC** at 10 μM (Fig. 3a), corresponding to a visual color change from yellow to orange red. Concurrent with the absorption change, dramatic changes of the PL spectra were observed upon TFA addition (Fig. 3c). The emission peak at 464 nm was gradually quenched with increasing amount of TFA. Meanwhile, a new peak centred at 523 nm after saturation emerged at a longer wavelength, corresponding to a change of fluorescence from blue to yellow. The PL spectroscopic changes suggest that the **ODP-BAC** can be used as a dual-band fluorescence sensor by monitoring the fluorescence “turn-off”at 464 nm and fluorescence “turn-on” at 523 nm. Such a dual-band sensing should increase the reliability and specificity of proton sensing by minimizing the false positive.^15^ Plotting the absorption increase at 484 nm and PL decrease at 464 nm against the amount of TFA indicated near linear relationships in the low concentration range (Fig. 3b and d), which would allow for quantitative data analysis for determining the amount of acid present.

**Fig. 4:**
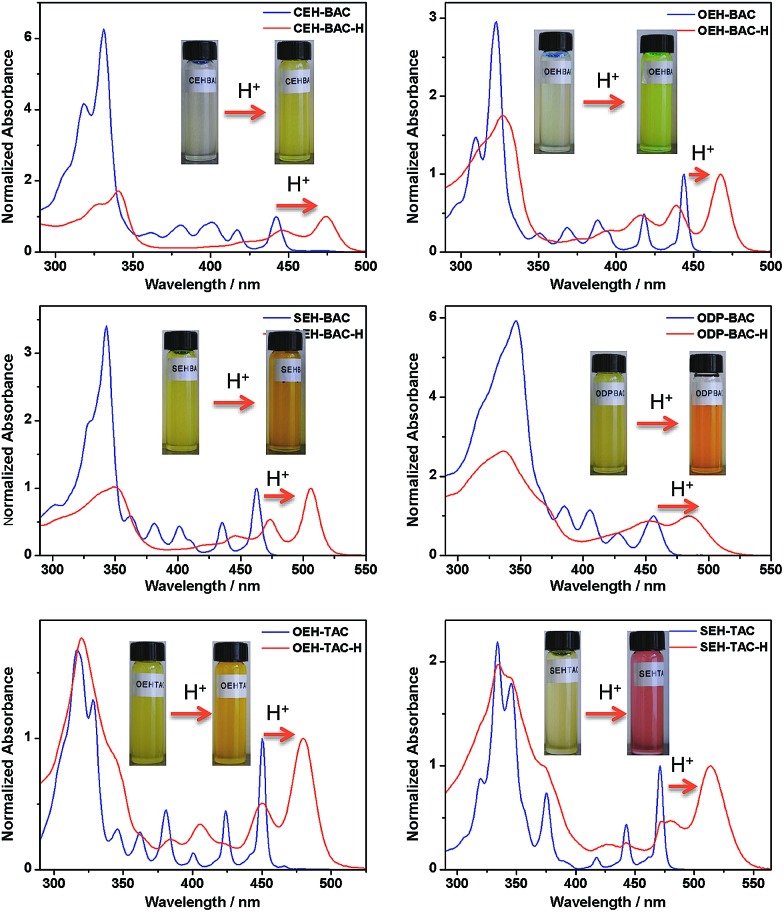
Acid-induced UV-vis absorption changes of azacoronenes. Insert: photographs showing the visual color changes of azacoronenes under ambient light.

**Fig. 5:**
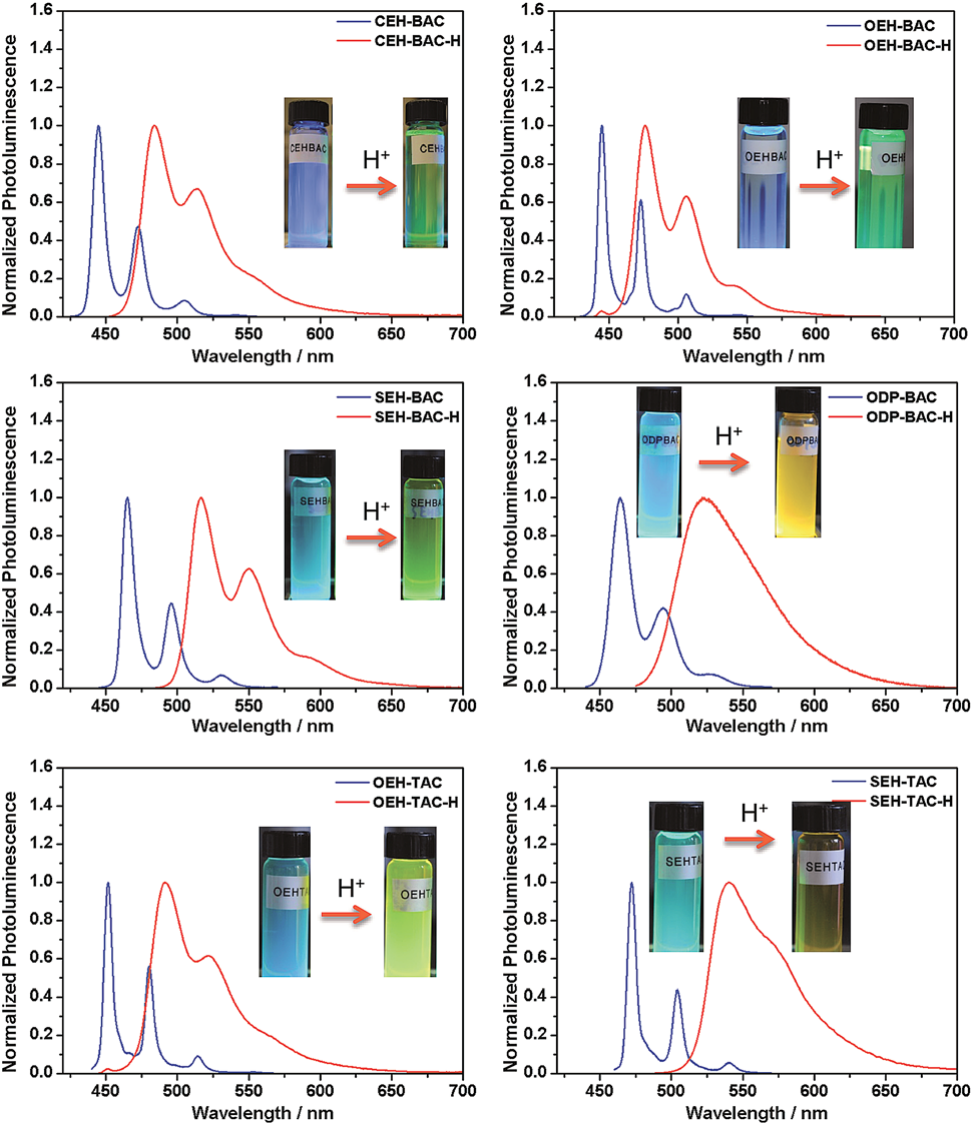
Acid-induced photoluminescence changes of azacoronenes. Insert: photographs showing the visual fluorescence color changes of azacoronenes under a 365 nm UV lamp.

Similar acid-induced changes of absorption and PL were observed for all the azacoronene derivatives. The addition of TFA provoked significant bathochromic shifts of the lowest energy absorption peaks in the range of 23 to 43 nm, accompanied by visual color changes (Fig. 4). Concurrent red shift of the emission peaks of up to 81 nm was also observed (Fig. 5 and S3†). QYs were in general slightly decreased upon protonation with the exception of **OEH-TAC** and **ODP-BAC** (Table 1). These experiments clearly suggest that the azacoronene family can function as “naked-eye” dual-mode probes for pH sensing applications, such as gamma-radiation sensors as recently reported by Zang *et al.*,^15^ with the detection wavelength of interest covering a wider spectrum. The acid responsiveness presumably originates from complexation between TFA and the basic N atoms on the azacoronene core, which correlates well with ^1^H NMR spectroscopic studies. As shown in Fig. S4,† the addition of TFA into the solution of **OEH-BAC** causes the up-field shift of the pyridine protons, suggesting the formation of TFA complexes.

### Theoretical modeling of optical and electronic structures

Density functional theory (DFT) calculations on the nature of the molecular orbitals and the frontier orbital energies are carried out on **BAC** and **TAC** derivatives with different peripheral functional groups. A linear correlation correction (eqn (1))^16^ is applied for the calculated HOMO and LUMO eigenenergies including toluene solvent^17^ on account of the systematic overestimation by B3LYP functional.^18^
1
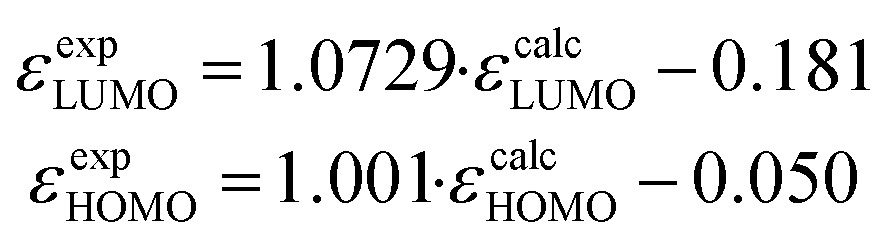



**Fig. 6:**
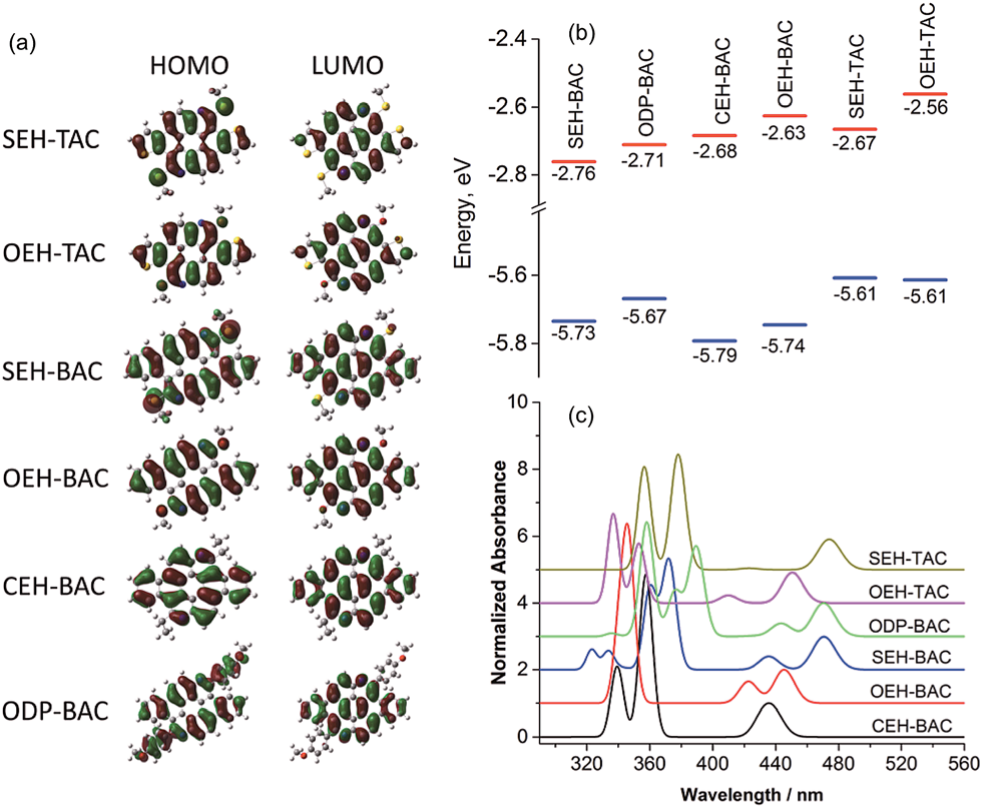
(a) Frontier orbitals, (b) correlation diagram of HOMO (blue) and LUMO (red) orbital energies, and (c) calculated absorption spectra of the **TAC** and **BAC** derivatives.

As illustrated in Fig. 6a, the LUMO orbitals in all **BAC** and **TAC** derivatives are highly delocalized over the core, while the contribution from the peripheral groups is negligible. According to the ligand field theory, the more electron-withdrawing a functional group is, the more the HOMO is stabilized and the LUMO is destabilized. Since localization of LUMO orbitals is very similar for all **BAC** molecules, the order of LUMO energies (Fig. 6b) reflects the relative electron withdrawing strength of the peripheral groups, which is in the following order: alkylsulfide > phenyl > alkyl > alkoxy. As far as the HOMOs are concerned, the HOMO orbital of **ODP-BAC** is delocalized over the core unit as well as the peripheral phenyl rings, resulting in increased HOMO energy. Alkylsulfide and alkoxy destabilizes the HOMO to a very similar extent, while the alkyl group gives the lowest HOMO energy levels.

For **BAC** and **TAC** derivatives bearing the same peripheral groups, the calculated bandgaps are slightly smaller for **TAC** derivatives, which are consistent with the experimental observations. The **TAC** core is more electron rich than the **BAC** one due to the introduction of more electron rich thiophene unit, as reflected by the higher HOMO and LUMO energy levels for both alkoxy and alkylsulfide substitutions. It is worth noting that in both **BAC** and **TAC** cases, the alkylsulfide and alkoxy groups possess the same HOMO but different LUMO energy levels. The lower LUMO energy level of alkylsulfide again confirms that it is more electron withdrawing than the alkoxy group. Such findings should be very valuable in guiding the fine tuning of optoelectronic properties of organic semiconductors.

The absorption spectra of these azacoronenes were also calculated (Fig. 6c) using toluene as the solvent. The experimental absorption spectra contain vibronic multiplet peaks at the longer wavelength region. On account of the overwhelming computational expenses, the vibronic effects were not included during the calculation and thus the vibronic features were not reproduced in the calculated spectra. Nevertheless, a good agreement is observed between theory and experiment in terms of band positions, relative intensities and overall shape. In particular, the measured (calculated) first absorption bands are 471 (462), 463 (459), 456 (459), 450 (442), 444 (435) and 442 (429) nm for **SEH-TAC**, **SEH-BAC**, **ODP-BAC**, **OEH-TAC**, **OEH-BAC** and **CEH-BAC**, respectively.

## Conclusions

We have reported a modular synthetic approach of bisaryl-annulated azacoronene series using **DAP** intermediates, which facilitate the alteration of both the core structure and peripheral heteroatoms of a versatile azacoronene PAH system. The α-chloropyridine moiety is compatible with aromatic substitution reactions, Kumada coupling or Suzuki coupling reactions, from which thioalkyl, alkoxy, alkyl, or aryl substituents are readily introduced to the benzo- or thienoazacoronene cores. Such a family of PAHs allows a structure–property relationship study as verified by both experiments and theoretical calculations. The heteroatom linkages not only impact the intercolumnar arrangement, but also greatly change the optoelectronic properties. The thin films of these azacoronenes show strong fluorescence, implying their great potential for use in solid-state light emitting applications. In addition, the azacoronenes display significant spectroscopic responses when protonated, clearly suggesting that they can function as “naked-eye”dual-mode probes for protons. The synthetic methodology thus represents a concise route to an array of novel colorimetric and fluorescence pH sensors with tunable spectroscopic ranges.

## Supplementary Material

Supplementary information

Crystal structure data
