# Supplementary material for: A divergent route to core- and peripherally functionalized diazacoronenes that act as colorimetric and fluorescence proton sensors
Source: Chem Sci. 2015 Mar 31;6(5):3180–6. doi: 10.1039/c5sc00304k (PMC5657404; doi:10.1039/c5sc00304k)
Supplement: Supplementary file 1 [file SC-006-C5SC00304K-s001.pdf]

Electronic Supplementary Material (ESI) for Chemical Science.
This journal is © The Royal Society of Chemistry 2015

Supporting Information

A Divergent Route to Core- and Peripherally Functionalized Diazacoronenes that Act As Colorimetric and Fluorescent Proton Probes

Bo He,[†a] Jing Dai,[†b] Danylo Zherebetskyy,[c] Teresa L. Chen,[a] Benjamin A. Zhang,[a] Simon J. Teat,[d] Qichun Zhang,[e] Linwang Wang[c] and Yi Liu[a]*

[a]The Molecular Foundry, [c]Materials Sciences Division, and [c]Advanced Light Source, Lawrence Berkeley National Laboratory, One Cyclotron Road, Berkeley, California, 94720, USA, [b]Department of Chemistry, Zhejiang University, 310027, Hangzhou, China, [e]School of Materials Science and Engineering, Nanyang Technological University, 50 Nanyang Avenue, 639798, Singapore.

Materials and Methods. Reagents were purchased from Aldrich or synthesized as described. Compound **3b** and **4b** were synthesized as previously described.[1] 2-(4-((2-octyldodecyl)oxy)phenyl)- 4,4,5,5- tetramethyl-1,3,2-dioxaborolane[2] and 2-decyltetradecyl-1-thiol[3] were synthesized according to literature. All other compounds were synthesized according to following synthetic procedures. Dry solvents were collected from a solvent purification system. Thin-layer chromatography (TLC) was carried out using aluminum sheets, precoated with silica gel 60F (Merck 5554). The plates were inspected by UV-light. Proton and carbon nuclear magnetic resonance spectra (^{1}H-NMR and ^{13}C-NMR) were recorded on a Bruker Avance500 II, using the deuterated solvent as lock and tetramethylsilane as internal standard. All chemical shifts are quoted using the δ scale, and all coupling constants (J) are expressed in Hertz (Hz). Matrix-assisted laser desorption ionization (MALDI) mass spectra were measured on 4800 MALDI TOF/TOF analyzer from Applied Biosystems. UV-Vis-NIR spectra were recorded using a Cary 5000 UV-Vis-NIR spectrometer. Photoluminescence spectrua were recorded using a HORIBA JOBIN YVON fluorimeter. Cyclic voltammetry was performed using a 273A potentiostat (Princeton Applied Research), wherein glassy carbon, platinum and a silver wire act as the working electrode, the counter electrode and the pseudo-reference electrode, respectively. Thin film samples were prepared by dropcasting the $CHCl_3$ solutions onto the working electrodes, with tetrabutylammonium hexafluorophosphate (0.1 M) as the electrolyte at a scan rate of 100 mV s^{-1}, using ferrocene/ferronium (Fc/Fc^+) redox couple as an internal standard. The HOMO and LUMO levels of compounds are calculated from the difference between the first oxidation potential (E_{oxi}) or reduction potential (E_{red}) of the compounds and the oxidation potential of ferrocene ($E_{HOMO} = -(4.8-E_{oxi})$ eV, $E_{LUMO} = -(4.8-E_{red})$ eV).

Crystallography information: Crystallographic information files (CIFs) were deposited in Cambridge structural database, with Cambridge Crystallographic Data Center (CCDC) numbers 1031859, 1031861, and 1032434 for compounds **SEH-BAC**, **SEH-TAC** and **OEH-BAC**, respectively. Crystals of **OEH-BAC**, **SEH-TAC** and **SEH-BAC** were mounted in the 100(2) K nitrogen cold stream provided by an Oxford Cryostream low temperature apparatus on a Bruker D85 diffractometer equipped with an ApexII CCD detector for **OEH-BAC** and with a PHOTON100 CMOS detector for **SEH-TAC** & **SEH-BAC**, on beamline 11.3.1 at the Advanced Light Source in Berkeley, CA. Diffraction data were collected using synchrotron radiation monochromated with silicon(111) to a wavelength of 0.77490(1) Å. A full sphere of data was collected using 0.3° ω scans for **OEH-BAC** and 1° ω scans, in shutterless mode for **SEH-TAC** & **SEH-BAC**. A multi-scan absorption correction was applied using the program SADABS. The data consists of: (**OEH-BAC)** 33486 reflections collected, of which 4164 were unique [R(int) = 0.0450] and 3412 were observed [I>2σ (I)] ; (**SEH-TAC**) 46645 reflections collected, of which 5560 were unique [R(int) = 0.0475] and 4614 were observed [I>2σ (I)]; (**SEH-BAC**) 16462 reflections collected, of which 2388 were unique [R(int) = 0.0592] and 1666 were observed [I>2σ (I)]. The structure was solved by intrinsic phasing (SHELXT) and refined by full-matrix least-squares on F^2 (SHELXL) using 287 parameters and 158 restraints; 245 parameters and 178 restraints; and 247 parameters and 38 restraints for **OEH-BAC**, **SEH-TAC** & **SEH-BAC** respectively. In all cases the alkyl chains were found to be disordered. Two orientations were identified and refined. Due to the overlapping of the two orientations, distance restraints were used to control the geometries in **OEH-BAC** & **SEH-BAC** and displacement parameter restraints were used in all cases. For **SEH-TAC** & **SEH-BAC** all hydrogen atoms were

generated geometrically and refined as riding atoms with C-H = 0.95- 0.99 Å and U_{iso}(H) =1.2 times U_{eq}(C) for CH and CH_2 groups and U_{iso}(H) =1.5 times U_{eq}(C) for CH_3 groups. For **OEH-BAC** the alkyl hydrogen atoms were treated as above while the aryl hydrogen atoms were found in the difference map and allowed to refine freely. The maximum and minimum peaks in the final difference Fourier map were 0.684 and -0.427 e.$Å^{-3}$; 0.256 and -0.260 e.$Å^{-3}$; 0.256 and -0.260 e.$Å^{-3}$; respectively for **OEH-BAC**, **SEH-TAC** & **SEH-BAC**. Crystal data **OEH-BAC**: $C_{42}H_{42}N_2$ O_2 S_2, Mw= 670.89, monoclinic, $P2_1/c$, a = 18.917(2) Å, b = 5.0175(6) Å, c = 17.6728(19) Å, β=95.137(6)°., V= 1670.7(3) $Å^3$, T= 100(2) K, Z= 2, R1 [I>2σ (I)] = 0.0550, wR2 (all data) = 0.1529, GOF (on F^2) = 1.043. Crystal data **SEH-TAC**: $C_{46}H_{46}N_2$ S_2, Mw= 690.97, monoclinic, $P2_1/c$, a = 23.6399(19) Å, b = 5.2070(3) Å, c = 15.0420(10) Å, β=107.545(5)°, V= 1765.4(2) $Å^3$, T= 100(2) K, Z= 2, R1 [I>2σ (I)] = 0.0661, wR2 (all data) = 0.1749, GOF (on F^2) = 1.084. Crystal data **SEH-BAC**: $C_{46}H_{46}N_2$ S_2, Mw= 690.97, monoclinic, $P2_1/c$, a = 23.6399(19) Å, b = 5.2070(3) Å, c = 15.0420(10) Å, β=107.545(5)°, V= 1765.4(2) $Å^3$, T= 100(2) K, Z= 2, R1 [I>2σ (I)] = 0.0661, wR2 (all data) = 0.1749, GOF (on F^2) = 1.084.

Computational Details

Calculations have been performed utilizing the density functional theory (DFT) at the B3LYP level of theory[4-5] as implemented Gaussian 09 package.[6] Inclusion of solvation effect is important when describing electronic structure and adsorption spectra.[7-9] Therefore, we computationally implemented toluene solvent by using continuum solvation model (SMD), which is based on the quantum mechanical charge density of a solute.[10] SMD shows reliable results for prediction of the excitation energies.[9] Ground state geometries of the molecules in the solvent have been optimized using 6-31+G(d) [Binkley1980, McLean1980] basis set.[11-12] Afterwards, the optimized geometries were used for excited state calculations relying on time-dependent DFT (TD-DFT) calculations using 6-311++G(d,p) basis set in the solvent. The simulations of the absorption spectra were performed by Gaussian functions convolution centered at the calculated excitation energies, with a half-width of 70 meV. Since the linear correction to the frontier orbitals have been applied, we applied corresponding scaling to the absorption spectra, which also provided better correspondence to the experimental measurements.

Experimental Section

Scheme S1. Synthesis of **DAPs**

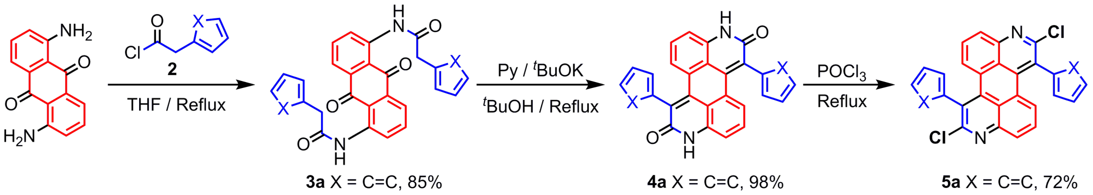

3a X = C=C, 85%
3b X = S, 92%
4a X = C=C, 98%
4b X = S, 83%
5a X = C=C, 72%
5b X = S, 93%

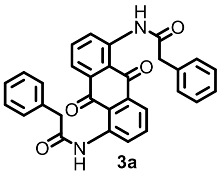

3a

3a. 1,5-diaminoanthraquinone (**1**, 10.0 g, 42.0 mmol, 1.0 eq) was first dissolved in anhydrous THF

(150 mL) under nitrogen. After phenylacetyl chloride (13.6 g, 11.7 mL, 88.1 mmol, 2.1 eq) was added dropwise into the stirring solution, the mixture was heated to reflux for 24 h. After cooling down, the mixture was poured into water and the precipitate was filtered and washed with methanol. The title compound was collected as orange solid (17.0 g, 85%). ^{1}H NMR ($CDCl_3$, 500 MHz): δ = 12.27 (s, 2H) 9.15 (dd, *J* = 8.6 Hz, 1.1Hz, 2H), 7.98 (dd, *J* = 7.7 Hz, 1.1 Hz, 2H), 7.76 (t, *J* = 8.1 Hz, 2H), 7.45 (m, 8H), 7.38 (t, *J* = 7.2 Hz, 2H), 3.87 (s, 4H). ^{13}C NMR ($CDCl_3$, 125 MHz): δ = 186.33, 171.14, 141.76, 135.79, 134.37, 133.98, 129.61, 129.04, 127.56, 126.08, 122.68, 117.10, 46.10. MS (MALDI-TOF) for $C_{30}H_{22}N_2O_4$: 474.19 $[M]^+$.

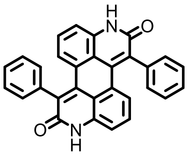

4a

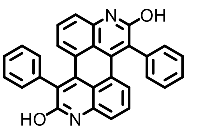

4a'

4a/4a'. A stirring solution of **3a** (15.0 g, 31.6 mmol, 1.0 eq) and *tert*-BuOK (14.2 g, 126.5 mmol, 4.0 eq) in *tert*-BuOH (300 mL) was first purged with nitrogen, followed by the addition of pyridine (30.02 g,30.56 mL, 37.93 mmol, 12.0 eq) at room temperature. The mixture was then stirred overnight at 105 ^{o}C. After cooling down, the mixture was quenched by saturated aqueous NH_4Cl solution until pH reached about 6. The resulting precipitate was collected after washing with methanol to give the title compound as red solid (13.6 g, 98%). Two sets of peaks were found in the ^{1}H NMR spectra (Figure S7), suggesting a mixture of two compounds. However, only one molecular weight as 438.13 was found in MALDI-TOF spectra. It was noticed that the chemical shift of –CH_2- in **3a** around 4 ppm disappeared in the NMR spectrum of **4a**, implying a complete condensation reaction. Based on these observations, the product was assigned to a mixture of **4a** and its isomer form **4a'** due to tautomerization during the base induced Knoevenagel type condensation reaction. This proposal was supported by the further neat transformation of **4a** to **5a** in the next step.

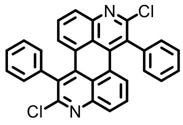

5a

5a. A mixture of **4a** (5.00 g, 13.1 mmol, 1.0 eq) and $POCl_3$ (50 mL) was heated to reflux overnight under N_2 protection. After cooled down to room temperature, the resulting mixture was carefully poured into ice water to give the crude product as a precipitate. After filtration, the crude product was further purified by Soxhlet extraction with chloroform. The title compound was obtained as dark green solid (3.70 g, 72% yield). ^{1}H NMR ($CDCl_3$, 500 MHz): δ = 7.93 (dd, *J* = 8.1 Hz, 1.2 Hz, 2H), 7.59 (m, 6H), 7.43 (m, 4H), 7.38 (dd, *J* = 7.8 Hz, 1.2 Hz 2H), 7.32 (t, *J* = 8.0 Hz, 2H). ^{13}C NMR ($CDCl_3$, 125 MHz): δ = 153.17, 146.98, 138.88, 138.39, 130.93, 130.29, 130.10, 130.04, 129.89, 129.44, 128.75, 128.44, 124.95. MS (MALDI-TOF) for $C_{30}H_{16}Cl_2N_2$: 474.06 $[M]^+$.

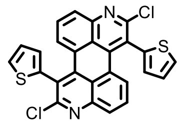

5b

5b. A mixture of **4b** (2.00 g, 4.44 mmol, 1.0 eq) and $POCl_3$ (20 mL) was heated to reflux

overnight under N_2 protection. After cooled down to room temperature, the resulting mixture was carefully poured into ice water to give the crude product as a precipitate. After filtration, the crude product was further purified by Soxhlet extraction with chloroform. The title compound was obtained as dark green solid (2.01 g, 93% yield).). ^{1}H NMR ($CDCl_3$, 500 MHz, 298 K): δ = 8.00 (dd, J = 8.3 Hz, 1.0 Hz, 2H), 7.63 (m, 4H), 7.48 (t, J = 8.1 Hz, 2H), 7.29 (dd, buried in $CHCl_3$, 2H), 7.24 (m, 2H). ^{13}C NMR ($CDCl_3$, 125 MHz): δ = 153.82, 147.12, 140.04, 139.32, 130.48, 130.11, 129.71, 129.33, 128.58, 128.51, 128.29, 124.65, 123.64. MS (MALDI-TOF) for $C_{26}H_{12}Cl_2N_2S_2$: 485.98 $[M]^+$.

Scheme S2. Synthesis of **BAC**s and **TAC**s

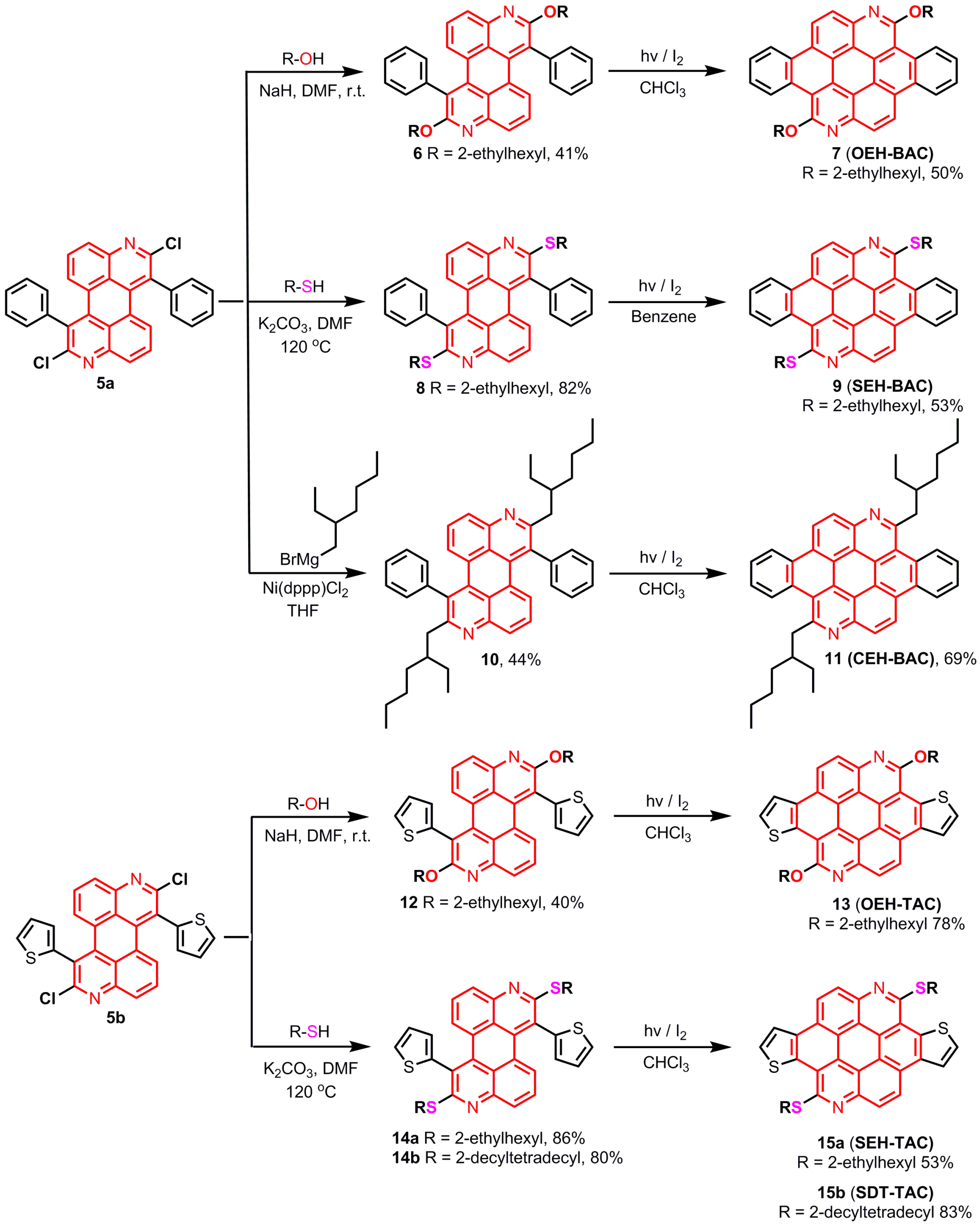

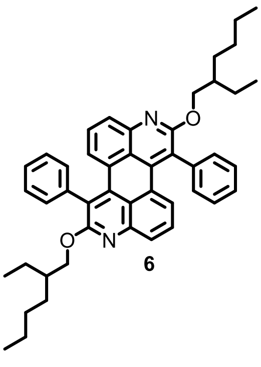

6

6. A mixture of **5a** (2.00 g, 4.21 mmol, 1.0 eq) and NaH (303 mg, 12.6 mmol, 3.0 eq) was purged with nitrogen, followed by the addition of anhydrous DMF (30 mL). 2-Ethylhexan-1-ol (1.37 g, 10.5 mmol, 2.5 eq) was added at 0 ^{o}C. The mixture was stirred at room temperature overnight and then poured into ice water. The yellow solid was collected by filtration and purified via silica gel column chromatography (pure hexanes to 4:1 of hexanes/chloroform). The title compound was isolated as yellow solid (1.14 g, 41%). ^{1}H NMR ($CDCl_3$, 500 MHz): δ = 7.70 (dd, *J* = 8.1 Hz, 1.1 Hz, 2H), 7.43 (m, 6H), 7.26 (d, *J* = 1.2 Hz, 2H), 7.20 (t, *J* = 8.0 Hz, 2H), 4.34 (d, *J* = 5.4 Hz, 24H), 1.61 (m, 2H), 1.31-1.25 (m, 8H), 1.23 (m, 8H), 0.86 (m, 12H). ^{13}C NMR ($CDCl_3$, 125 MHz): δ = 161.22, 146.00, 138.22, 137.45, 130.17, 129.15, 128.93, 128.12, 127.72, 127.43, 127.21, 123.25, 123.12, 68.44, 39.04, 30.66, 28.96, 24.05, 23.04, 14.09, 11.20. MS (MALDI-TOF) for $C_{46}H_{50}N_2O_2$: 663.41 $[M]^+$.

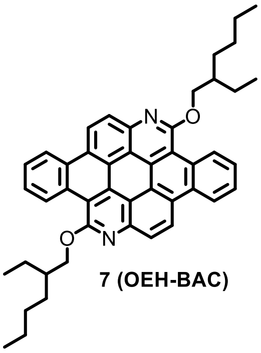

7 (OEH-BAC)

7 (OEH-BAC). A solution of **6** (0.50 g, 0.75 mmol, 1.0 eq) in chloroform (30 mL) was irradiated with a 300W incandescent lamp with catalytic amount of iodine for 24 h. After removal of the solvent under reduced pressure, the crude product was isolated via silica gel column chromatography (hexanes/chloroform 1:1) to give a yellow solid, which was further purified by recrystallization from cyclohexane to give a yellow crystal (0.25 g, 50%). ^{1}H NMR ($CDCl_3$, 500 MHz): δ = 10.0 (m, 2H), 9.02 (d, *J* = 9.5 Hz, 2H), 8.96 (dd, *J* = 6.4 Hz, 2.7 Hz, 2H), 8.36 (d, *J* = 8.9 Hz, 2H), 7.89 (m, 4H), 4.91 (dd, *J* = 5.6 Hz, 3.2 Hz, 4H), 2.22 (m, 2H), 1.93-1.72 (m, 8H), 1.65 (m, 4H), 1.54 (m, 4H), 1.22 (t, *J* = 7.5 Hz, 6H), 1.06 (t, *J* = 7.3 Hz, 6H). ^{13}C NMR ($CDCl_3$, 125 MHz): δ = 159.54, 140.91, 129.16, 128.65, 128.34, 127.22, 126.99, 126.14, 126.07, 125.66, 122.75, 122.46, 119.65, 114.39, 111.27, 69.57, 39.44, 31.36, 29.48, 24.66, 23.33, 14.36, 11.61. MS (MALDI-TOF) for $C_{46}H_{46}N_2O_2$: 658.35$[M]^+$.

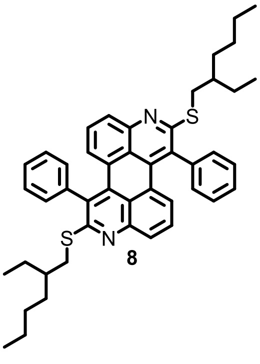

8. A mixture of **5a** (2.00 g, 4.21 mmol, 1.0 eq), K_2CO_3 (2.33 g, 16.8 mmol, 4.0 eq), 2-ethylhexyl-1-thiol (1.85 g, 2.19 mL, 12.6mmol, 3.0 eq) and 18-crown-6 (catalytic amount) were purged with nitrogen, followed by the addition of anhydrous DMF. The mixture was heated at 120°C overnight and then cooled to room temperature. After excess K_2CO_3 was removed by filtration and rinsed with chloroform, the combined filtrate was concentrated under reduced pressure to give a crude product, which was purified via silica gel column chromatography (pure hexanes to 4:1 hexanes/chloroform). The title compound was isolated as yellow solid (2.38 g, 82% yield). ^{1}H NMR ($CDCl_3$, 500 MHz): δ = 7.80 (dd, *J* = 8.0 Hz, 1.2 Hz, 2H), 7.60 (m, 6H), 7.39 (dd, *J* = 7.6 Hz, 1.6 Hz,4H), 7.18 (m, 4H), 3.34 (m, 4H), 1.74 (m, 2H), 1.56-1.47 (m, 4H), 1.43 (m, 4H), 1.40-1.35 (m, 4H), 1.00 (t, *J* = 7.4 Hz, 6H), 0.97 (t, *J* = 7.1 Hz, 6H). ^{13}C NMR ($CDCl_3$, 125 MHz): δ = 162.05, 147.34, 139.05, 134.24, 130.43, 130.41, 129.92, 128.79, 128.63, 128.60, 128.20, 127.88, 123.65, 39.13. 35.29, 32.87, 29.07, 26.01, 14.24, 11.10. MS (MALDI-TOF) for $C_{46}H_{50}N_2S_2$: 695.42 $[M]^+$.

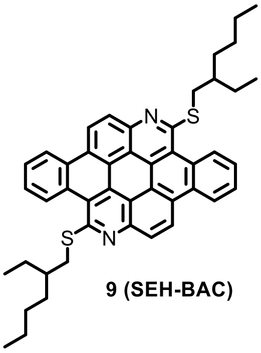

9 (SEH-BAC): Synthesis of **SEH-BAC** was similar to that of **OEH-TAC**. A solution of **8** (0.50 g, 2.83 mmol, 1.0 eq) in benzene (100 mL) was irradiated by a 300W incandescent lamp with catalytic amount of iodine for 48 h. After removal of the solvent under reduced pressure, the crude product was isolated via silica gel column chromatography (hexanes/chloroform 1:1) to give a yellow solid, which was further purified by recrystallization from toluene to give yellow crystal (258 mg, 53% yield). ^{1}H NMR ($CDCl_3$, 500 MHz): δ = 10.0 (dd, *J* = 6.6 Hz, 3.3 Hz,2H), 9.22 (d, *J* = 9.4 Hz, 2H), 9.11 (dd, *J* = 6.4 Hz, 3.4 Hz, 2H), 8.57 (d, *J* = 8.9 Hz, 2H), 8.00 (dd, *J* = 6.5 Hz, 3.2 Hz, 4H), 3.70 (m, 4H), 1.94 (m, 2H), 1.74-1.64 (m, 8H), 1.57-1.50 (m.4H), 1.45-1.38 (m, 4H), 1.10 (t, *J* = 7.4 Hz, 6H), 0.99 (t, *J* = 7.3 Hz, 6H). ^{13}C NMR ($CDCl_3$, 125 MHz): δ = 158.13, 142.46, 128.59, 128.05, 127.66, 127.27, 127.13, 126.16, 123.80, 123.37, 120.64, 120.62, 120.51, 117.02, 115.76, 39.08, 36.79, 33.14, 29.19, 26.28, 23.18, 14.24, 11.20. MS (MALDI-TOF) for $C_{46}H_{46}N_2O_2$: 691.29$[M]^+$.

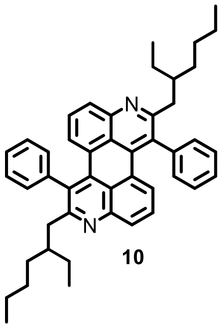

10

10: (2-Ethylhexyl)magnesium bromide (1.0 M, 0.52 mL, 0.52 mmol, 2.5 eq) was added to a stirring mixture of **5a** (100 mg, 0.21 mmol, 1 eq) and Ni(dppp)Cl_2 (5.7 mg, 0.01 mmol, 0.05 eq) in dry THF (20 mL) at 0 ^{o}C under an nitrogen atmosphere. The reaction mixture was first stirred at room temperature for 2h, followed by refluxing overnight. The reaction mixture was then cooled to 0 ^{o}C and quenched with aq. NH_4Cl. The mixture was extracted with $CHCl_3$ twice, the combined organic phase was washed with brine twice and dried over $MgSO_4$. After filtration, the solvent was removed under reduced pressure, and the resulting residue was subjected to silica gel column purification ($CHCl_3$/hexanes=8:1) to afford light yellow oil (58 mg, 44%). Ring closure reaction occurred during the purification process, resulting in a mixture of **10** and mono annulated PAH compound. The oil-like mixture was used directly for the following ring closure reaction without further purification.

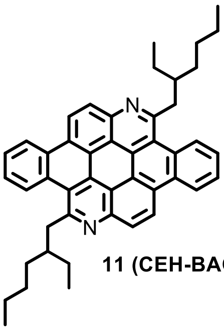

11 (CEH-BAC)

11. Synthesis of **11** was following the same synthetic procedure as that of **7**, using **10** and mono annulated **10** (55 mg) as the starting materials. **11** was obtained as light yellow solid (37 mg, 69%). ^{1}H NMR ($CDCl_3$, 500 MHz): δ = 9.48 (d, *J* = 9.3 Hz, 2H), 9.29 (t, *J* = 8.3 Hz, 4H), 8.90 (d, *J* = 8.9 Hz, 2H), 8.06 (t, *J* = 1.0 Hz, 2H), 7.99 (t, *J* = 1.0 Hz, 2H), 4.09 (d, *J* = 7.1 Hz, 4H), 2.55 (t, *J* = 5.7 Hz, 2H), 1.23-1.28 (m, 16H), 0.80 (m, 12H). ^{13}C NMR ($C_2D_2Cl_4$, 125 MHz, 60 ^{o}C): δ = 163.34, 133.97, 133.93, 133.33, 132.03, 131.99, 131.42, 131.20, 130.77, 129.86, 127.48, 126.52, 124.54, 123.94, 120.43, 49.87, 42.18, 42.12, 36.79, 36.73, 33.20, 32.42, 32.40, 29.97, 29.91, 26.50, 17.48, 17.42, 14.33. MS (MALDI-TOF) for $C_{46}H_{46}N_2$: 626.35$[M]^+$.

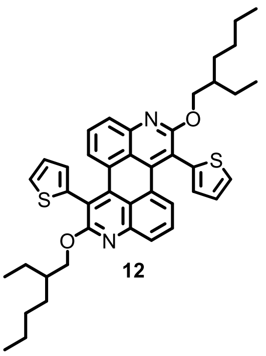

12

12. Synthesis of **12** was following the synthetic procedure of **6**. Yield: 40%. ^{1}H NMR ($CDCl_3$, 500 MHz): δ = 7.72 (dd, *J* = 8.1 Hz, 2.0 Hz, 2H), 7.51 (dd, *J* = 5.1 Hz, 1.1 Hz, 2H), 7.48 (dd, *J* = 7.8 Hz, 1.0 Hz, 2H), 7.32 (t, *J* = 8.0 Hz, 2H), 7.16 (dd, *J* = 5.1 Hz, 3.5 Hz, 2H), 7.08 (dd, *J* = 3.5 Hz, 1.1 Hz, 2H), 4.37(t, *J* = 4.8 Hz, 4H), 1.69 (m, 2H), 1.29-1.38 (m, 16H), 0.92 (m, 12H). ^{13}C NMR ($CDCl_3$, 125 MHz): δ = 161.18, 146.15, 139.28, 138.81, 128.72, 128.69, 128.11, 128.07, 127.74, 127.11, 126.68, 122.93, 115.72, 68.70, 39.09, 30.72, 29.04, 24.13, 23.09, 14.14, 11.26. MS (MALDI-TOF) for $C_{42}H_{46}N_2O_2S_2$: 674.54 $[M]^+$.

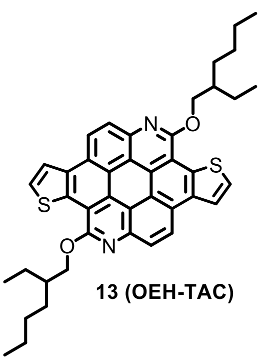

13 (OEH-TAC)

13. Synthesis of **13** was following the synthetic procedure of **7**. Yield: 40%. ^{1}H NMR ($CDCl_3$, 500 MHz): δ = 8.47 (d, *J* = 8.7 Hz, 2H), 8.23 (d, *J* = 8.6 Hz, 2H), 8.15 (d, *J* = 5.3 Hz, 2H), 7.93 (d, *J* = 5.3 Hz, 2H), 4.91 (m, 4H), 2.26 (m, 2H), 2.04 (m, 4H), 1.91 (m, 4H), 1.82 (m, 4H), 1.27 (t, *J* = 7.5 Hz, 6H), 1.09(t, *J* = 7.2 Hz, 6H). ^{13}C NMR ($CDCl_3$, 125 MHz): δ = 157.18, 139.37, 135.15, 131.19, 127.93, 126.90, 125.01, 124.2, 123.05, 121.29, 118.11, 114.21, 110.33, 69.60, 39.63, 31.02, 29.49, 24.43, 23.35, 14.37, 11.58. MS (MALDI-TOF) for $C_{42}H_{42}N_2O_2S_2$: 670.48 $[M]^+$.

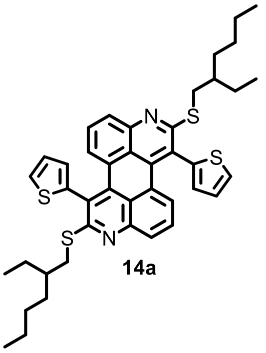

14a

14a. A mixture of **5b** (2.00 g, 4.10 mmol, 1.0 eq), K_2CO_3 (2.27 g, 16.4 mmol, 4.0 eq), 2-ethylhexyl-1-thiol (1.80 g, 2.13 mL, 12.3 mmol, 3.0 eq) and 18-crown-6 (catalytic amount) were purged with nitrogen, followed by the addition of dry DMF. The mixture was heated at 120 ^{o}C

overnight and then cooled to room temperature. After excess K_2CO_3 was removed by filtration and rinsed with chloroform, the combined filtrate was concentrated under reduced pressure to give a crude product, which was purified via silica gel column chromatography (pure hexanes to 4:1 hexanes/chloroform). The title compound was isolated as red oil (2.50 g, 86% yield). ^{1}H NMR ($CDCl_3$, 500 MHz): δ = 7.83 (dd, J = 8.1 Hz, 1.0 Hz, 2H), 7.61 (t, J = 4.1 Hz, 2H), 7.39 (d, J = 7.8 Hz, 2H), 7.33 (t, J = 8.0 Hz, 2H), 7.30 (d, buried in $CHCl_3$, 4H), 3.27 (m, 4H), 1.72 (m, 2H), 1.53-1.29 (m, 16H) 0.99 (t, J = 3.5 Hz, 6H), 0.95 (t, J = 5.4 Hz, 6H). ^{13}C NMR ($CDCl_3$, 125 MHz): δ = 163.14, 147.55, 139.49, 136.30, 129.74, 129.04, 128.90, 128.57, 128.28, 128.27, 127.30, 123.08, 122.81, 39.05, 35.31, 32.85, 29.03, 25.99, 23.06, 14.20, 11.07. MS (MALDI-TOF) for $C_{42}H_{46}N_2S_4$: 707.27 $[M]^+$.

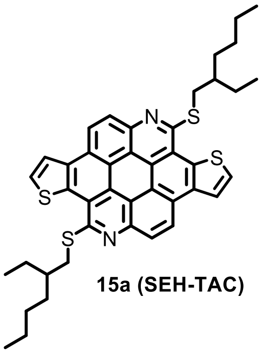

15a (SEH-TAC)

15a (SEH-TAC). Synthesis of **SEH-TAC** was similar to that of **OEH-TAC**. A solution of **10** (2.00 g, 2.83 mmol, 1.0 eq) in chloroform (100 mL) was irradiated with a 300W incandescent lamp with catalytic amount of iodine for 24 h. After removal of the solvent under reduced pressure, the crude product was isolated via silica gel column chromatography (hexanes/chloroform 1:1) to give a yellow solid, which was further purified by recrystallization from toluene to give yellow crystal (1.06 g, 53%). ^{1}H NMR ($CDCl_3$, 500 MHz): δ = 8.43 (d, J = 7.9 Hz, 2H), 8.14 (d, J = 5.1 Hz, 2H), 8.11 (d, J = 8.5 Hz, 2H), 7.90 (d, J = 5.2 Hz, 2H), 3.75-3.79 (m, 4H), 2.07 (m, 2H), 1.63-1.86 (m, 12H), 1.49-1.52 (m, 4H), 1.23 (t, J = 7.4 Hz, 6H), 1.08 (t, J = 7.3 Hz, 6H). ^{13}C NMR ($CDCl_3$, 125 MHz): δ 157.62, 150.06, 141.99, 137.69, 134.41, 129.54, 129.45, 126.52, 125.40, 125.11, 122.92, 119.96, 116.61, 41.11, 38.56, 34.84, 30.71, 28.10, 24.46, 15.40, 12.66. MS (MALDI-TOF) for $C_{42}H_{42}N_2S_4$: 703.16 $[M]^+$.

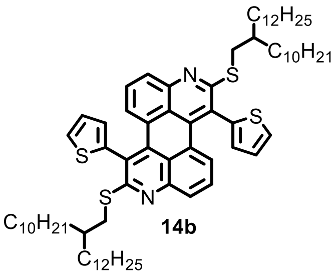

14b

14b. A mixture of **9** (1.31 g, 2.69 mmol, 1.0 eq), K_2CO_3 (1.49 g, 10.8 mmol, 4.0 eq), 2-decyltetradecyl-1-thiol (3.00 g, 8.09 mmol, 3.0 eq) and18-crown-6 ether (catalytic amount) were purged with nitrogen, followed by the addition of dry DMF. The mixture was heated to 120°C overnight and then cooled. After excess K_2CO_3 was removed by filtration and rinsed with chloroform, the filtrate was concentrated under reduced pressure to give a crude product, which was purified via silica gel column chromatography (pure hexanes to 6:1 hexanes/chloroform). The title compound was isolated as red oil (2.50 g, 80% yield). ^{1}H NMR ($CDCl_3$, 500 MHz): δ = 7.83

(dd, J =8.2Hz, 0.9 Hz, 2H), 7.60 (t, J = 3.5 Hz, 2H), 7.43 (d, J = 7.1 Hz, 2H), 7.34 (t, J = 8.0 Hz, 2H), 7.29 (d, buried in $CHCl_3$, 4H), 3.26 (d, J = 6.3 Hz, 4H), 1.75 (t, J = 5.5 Hz, 2H), 1.28-1.43 (m, 78H), 0.90 (m, 12H). ^{13}C NMR ($CDCl_3$, 125 MHz): δ = 163.24, 147.60, 139.51, 136.25, 129.73, 129.06, 128.90, 128.65, 128.27, 128.26, 127.31, 123.32, 122.87, 37.61, 35.78, 34.68, 33.60, 31.95, 31.61, 30.00, 29.74, 29.71, 29.70, 29.39, 26.73, 25.29, 22.71, 22.68, 14.15. MS (MALDI-TOF) for $C_{74}H_{110}N_2S_4$:1155.61 $[M]^+$.

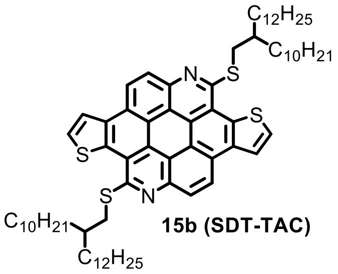

15b (SDT-TAC)

15b (SDT-TAC): Synthesis of **SDT-TAC** was similar to that of **OEH-TAC**. **11** (2.20 g, 1.90 mmol, 1.0 eq) in chloroform (100 mL) was irradiated with a 300W incandescent lamp with catalytic amount of iodine for 24 h. After the evaporation of solvent under reduced pressure, the crude product was isolated via silica gel column chromatography (hexane/chloroform = 1:1) to give a yellow solid, which was further purified by recrystallization from toluene to give yellow crystal (1.83g, 83% yield).^{1}H NMR ($CDCl_3$, 500 MHz): δ = 8.70 (d, J =8.8Hz, 2H), 8.40 (d, J = 8.7 Hz, 2H), 8.33 (d, J = 5.6 Hz, 2H), 8.01 (d, J = 5.4 Hz, 2H), 3.88 (d, J = 6.3 Hz, 4H), 2.11 (m, 2H), 1.79-1.65 (m, 20H), 1.41 (s, 18H), 1.27 (m, 42H), 0.87 (t, J = 6.9Hz, 12H). ^{13}C NMR ($CDCl_3$, 125 MHz, 50 ^{o}C): δ = 155.96, 140.42, 136.18, 132.85, 128.01, 127.88, 124.96, 123.83, 123.59, 121.49, 118.37, 115.02, 38.07, 37.38, 34.12, 31.89, 31.87, 30.15, 29.75, 29.72, 29.69, 29.67, 29.62, 29.32, 29.29, 27.03, 22.60, 13.95. MS (MALDI-TOF) for $C_{74}H_{106}N_2S_4$:1151.61 $[M]^+$.

Scheme S3. Synthesis of conjugation extended **BAC**.

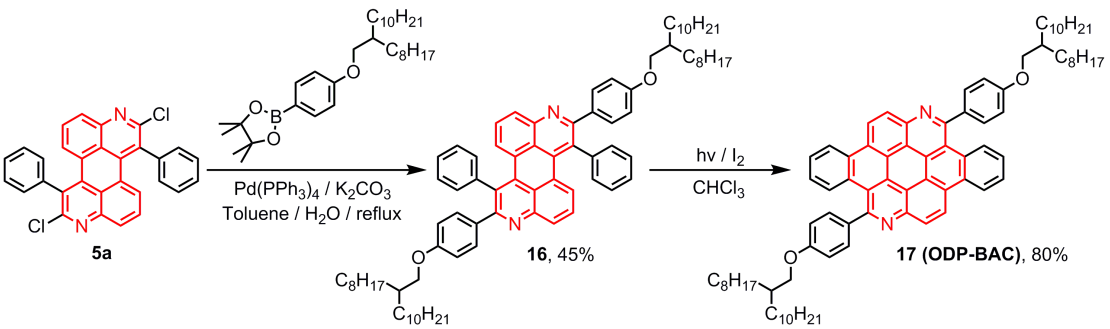

5a **16**, 45% **17 (ODP-BAC)**, 80%

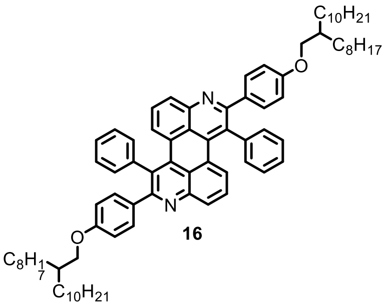

16

16. In a sealed microwave vial, a mixture of **5a** (500 mg, 1.05 mmol, 1 eq),

2-(4-((2-octyldodecyl)oxy)phenyl)-4,4,5,5-tetramethyl-1,3,2-dioxaborolane (1.21 g, 2.42 mmol, 2.3 eq), aq. K_2CO_3 (1.74 g, 6.31 mmol, 12 eq, 2M), $Pd(PPh_3)_4$ (36 mg, 0.03 eq) and one drop of Aliquat 336 in toluene was heated to 120 ^oC for 2 days. The crude product from general work-up of extraction and drying was purified by silica gel column with $CHCl_3$/hexanes (4:1) as eluent, to give the product as yellow solid (544 mg, 45%). ^1H NMR ($CDCl_3$, 500 MHz): δ = 7.96 (d, *J* = 7.5 Hz, 2H), 7.33-7.34 (m, 6H), 7.23 (d, *J* = 8.7 Hz, 4H), 7.19-7.22 (m, 4H), 7.17 (d, *J* = 8.0 Hz, 2H), 7.10 (dd, *J* = 7.5 Hz, 0.75 Hz, 2H), 6.79 (d, *J* = 8.8 Hz, 4H), 3.85 (d, *J* = 5.6 Hz, 4H), 1.81 (t, *J* = 5.7 Hz, 2H), 1.33 (m, 60H), 0.96 (m, 12H). ^{13}C NMR ($CDCl_3$, 125 MHz): δ = 160.31, 158.97, 146.87, 140.47, 136.26, 133.05, 131.06, 130.96, 130.81, 130.01, 129.51, 128.73, 128.27, 127.47, 124.88, 113.88, 70.94, 37.94, 32.01, 31.41, 30.11, 29.77, 29.69, 29.45, 29.43, 26.91, 22.78. MS (MALDI-TOF) for $C_{82}H_{106}N_2O_2$:1150.75 $[M]^+$.

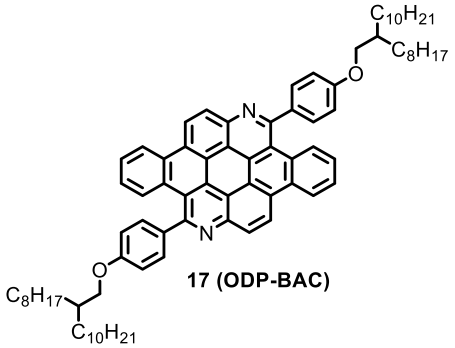

17 (ODP-BAC)

17. Synthesis of **17** was following the synthetic procedure of **7**. Yield: 80%. ^1H NMR ($CDCl_3$, 500 MHz): δ = 9.09 (d, *J* = 9.6 Hz, 2H), 8.98 (d, *J* = 8.1 Hz, 2H), 8.63 (d, *J* = 8.9 Hz, 2H), 8.39 (d, *J* = 8.3 Hz, 2H), 7.86-7.90 (m, 2H), 7.68 (d, *J* = 8.5 Hz, 4H), 7.55 (m, 2H), 7.14 (d, *J* = 8.7 Hz, 4H), 4.03 (d, *J* = 5.6 Hz, 4H), 1.921 (t, *J* = 5.8 Hz, 2H), 1.33-1.49 (m, 60H), 0.93 (m, 12H). ^{13}C NMR ($CDCl_3$, 125 MHz): δ = 159.95, 157.81, 142.35, 136.80, 130.72, 129.96, 129.66, 129.18, 128.16, 127.77, 127.64, 127.07, 125.45, 123.69, 123.41, 119.70, 118.80, 116.46, 115.33, 71.27, 38.04, 31.97, 31.45, 30.13, 29.76, 29.74, 29.71, 29.69, 29.41, 26.96, 22.73, 14.16. MS (MALDI-TOF) for $C_{82}H_{104}N_2O_2$:1146.75 $[M]^+$.

References

1. B. He, A. Pun, L. Klivansky, A. McGough, Y. F. Ye, J. F. Zhu, J. H. Guo, S. Teat, Y. Liu, *Chem. Mater.*, **2014**, *26*, 3920–3927.

2. M. Nuita, J. Sakuda, Y. Hirai, M. Funahashi, T. Kato, T.; *Chem. Lett.* **2011**, *40*, 412-413.

3. B. Tylleman, G. Gbabode, C. Amato, C. Buess-Herman, V. Lemaur, J. Cornil, R. G. Aspe, Y. H. Geerts, S. Sergeyev, *Chem. Mater.* **2009**, *21*, 2789.

4. A. D. Becke, *J. Chem. Phys.* **1993**, *98*, 5648-5652.

5. C. Lee, W. Yang, R. G. Parr, *Phys. Rev. B* **1988**, *37*, 785-789.

6. M. J. Friesh et al, *Gaussian 09*, revision A.2; Gaussian, Inc.: Wallingdorf, CT, 2009.

7. J.-F. Guillemoles, V. Barone, L. Joubert, C. Adamo, *J. Phys. Chem. A* **2002**, *106*, 11354-11360.

8. S. Fantacci, F. De Angelis, A. Selloni, A. *J. Am. Chem. Soc.* **2003**, *125*, 4381-4387.

9. S. Fleming, A. Mills, T. Tuttle, *Beilstein J. Org. Chem.* **2011,** *7,* 432–441.

10. A. V. Marenich, C. J. Ramer, D. G. Truhlar, *J. Phys. Chem. B* **2009,** *113,* 6378-6396.

11. J. S. Binkley, J. A. Pople, W. J. Hehre, *J. Am. Chem. Soc.* **1980**, *102*, 939-947.

12. R. Krishnan, J. S. Binkley, R. Seeger, J. A. Pople, *J. Chem. Phys.* **1980**, *72*, 650-654.

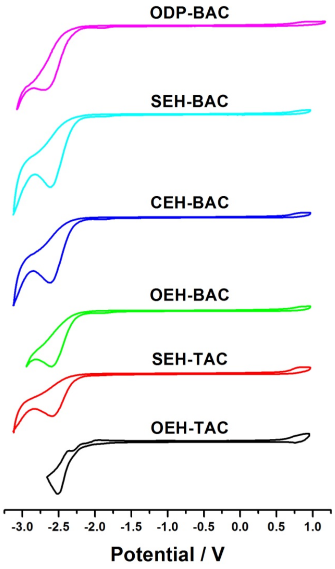

Sample	HOMO (film)	LUMO (film)	E_g^{elec} (film)
**OEH-BAC**	-5.39	-2.59	2.80
**SEH-BAC**	-5.38	-2.57	2.81
**CEH-BAC**	-5.42	-2.54	2.88
**OEH-TAC**	-5.42	-2.65	2.77
**SEH-TAC**	-5.42	-2.70	2.72
**ODP-BAC**	-5.44	-2.63	2.81

Figure S1. Left: Cyclic voltammetry of **BAC**s and **TAC**s (Scan rate: 100 mV/s). Right: HOMO, LUMO and Electrical gaps determined by CV.

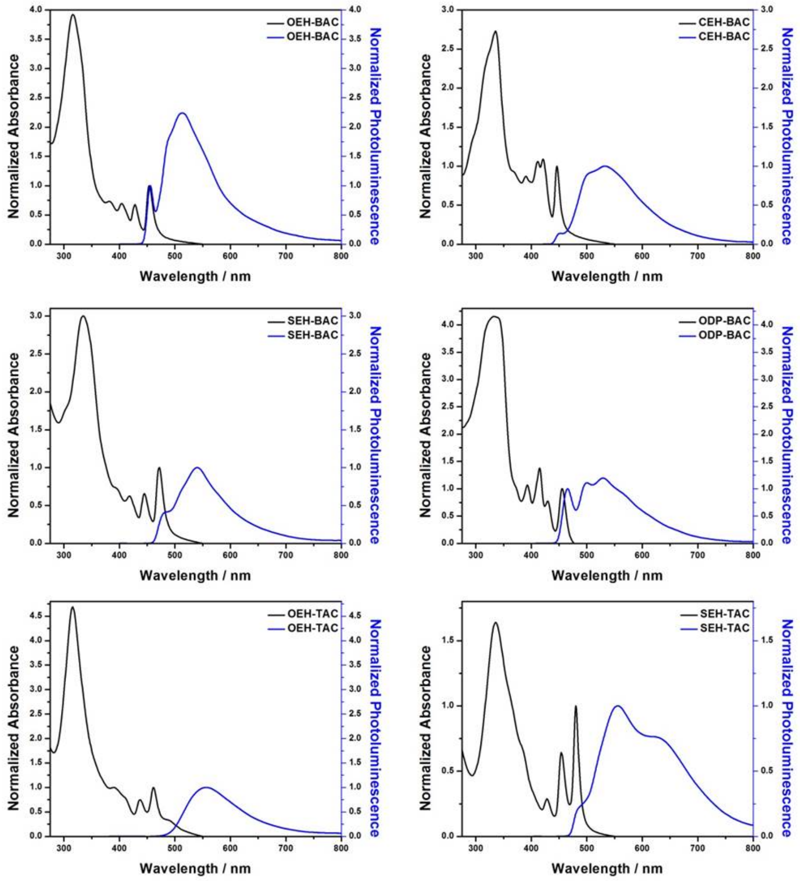

Figure S2. Normalized Absorption and PL spectra of **BAC**s and **TAC**s in thin films.

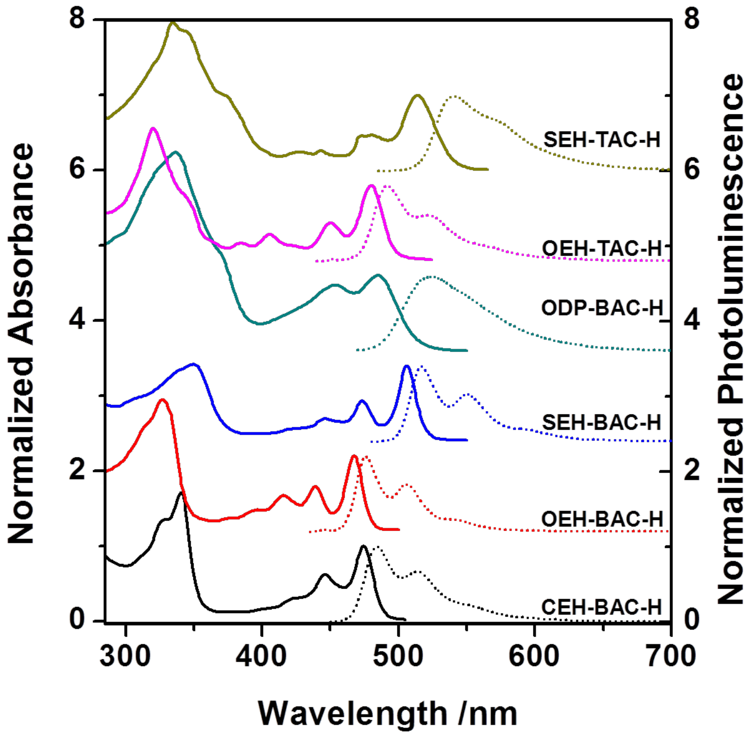

Figure S3. Normalized UV and PL spectra of protonated **BAC**s and **TAC**s.

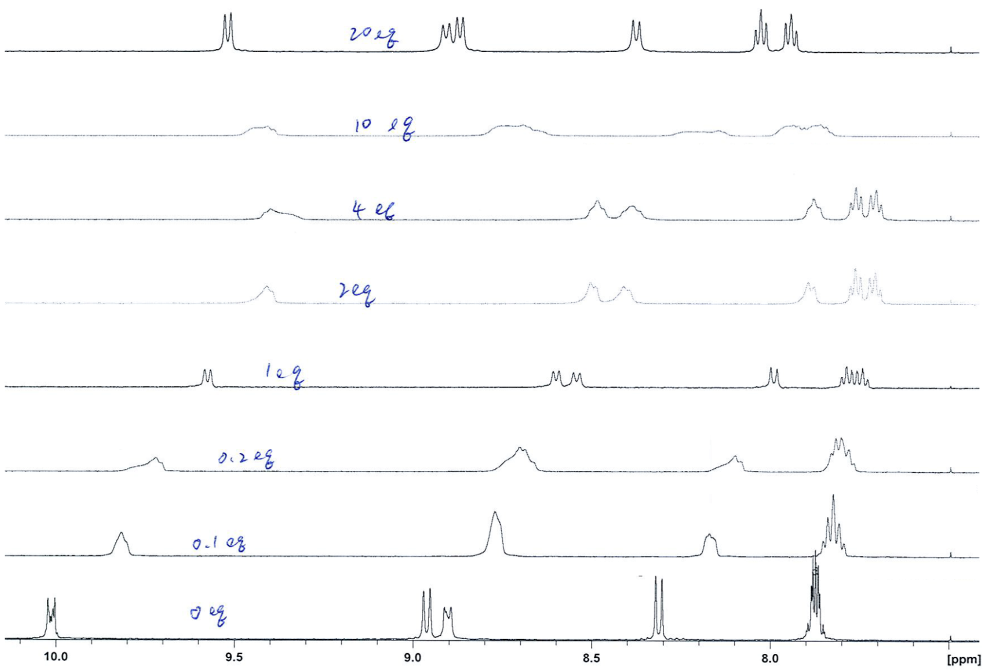

Figure S4. CF_3COOD-induced spectroscopic changes of the ^{1}H-NMR spectra of **OEH-BAC** ($CDCl_3$, 500 MHz, 298 K).

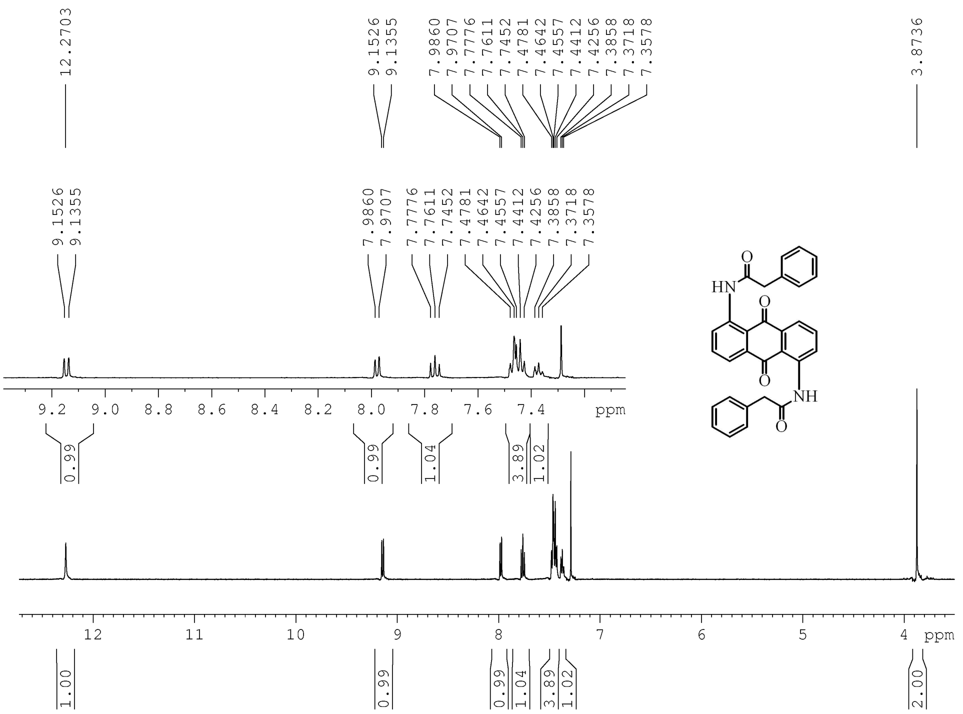

Figure S5. ^{1}H NMR spectrum of **3a** ($CDCl_3$, 500 MHz, 298K).

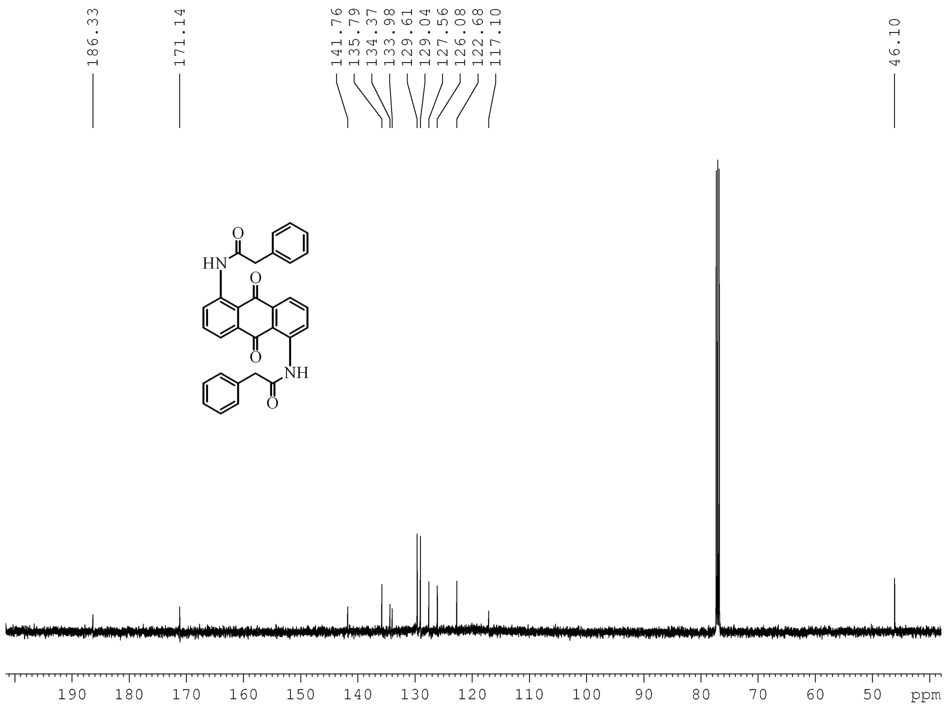

Figure S6. ^{13}C NMR spectrum of **3a** ($CDCl_3$, 125 MHz, 298K).

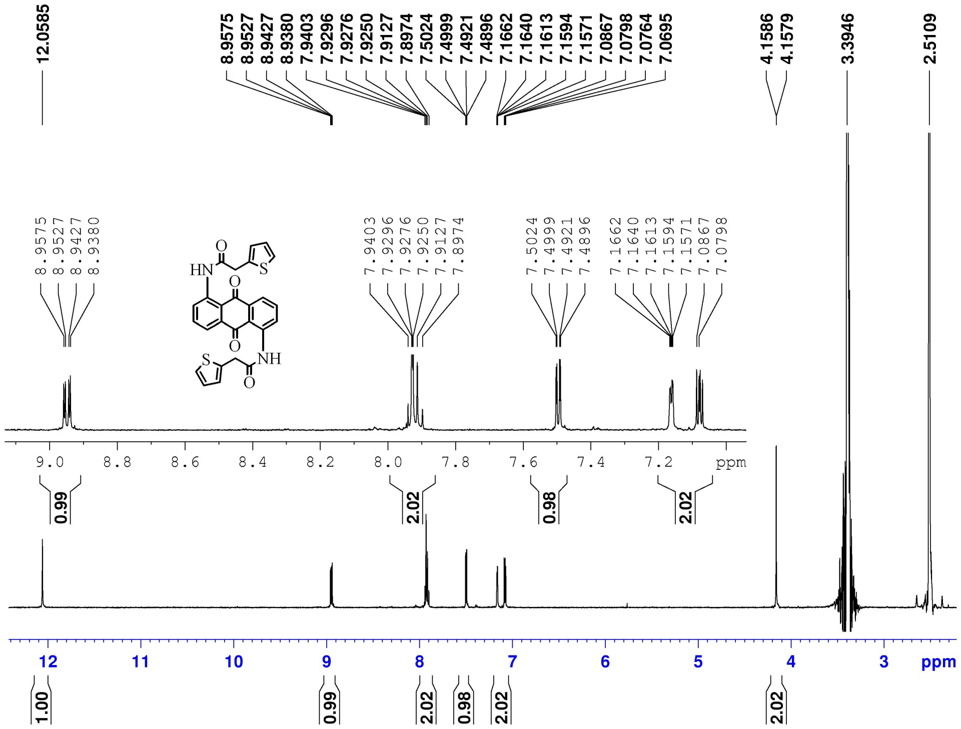

Figure S7. ^{1}H NMR spectrum of **3b** (DMSO-d_6, 500 MHz, 298K).

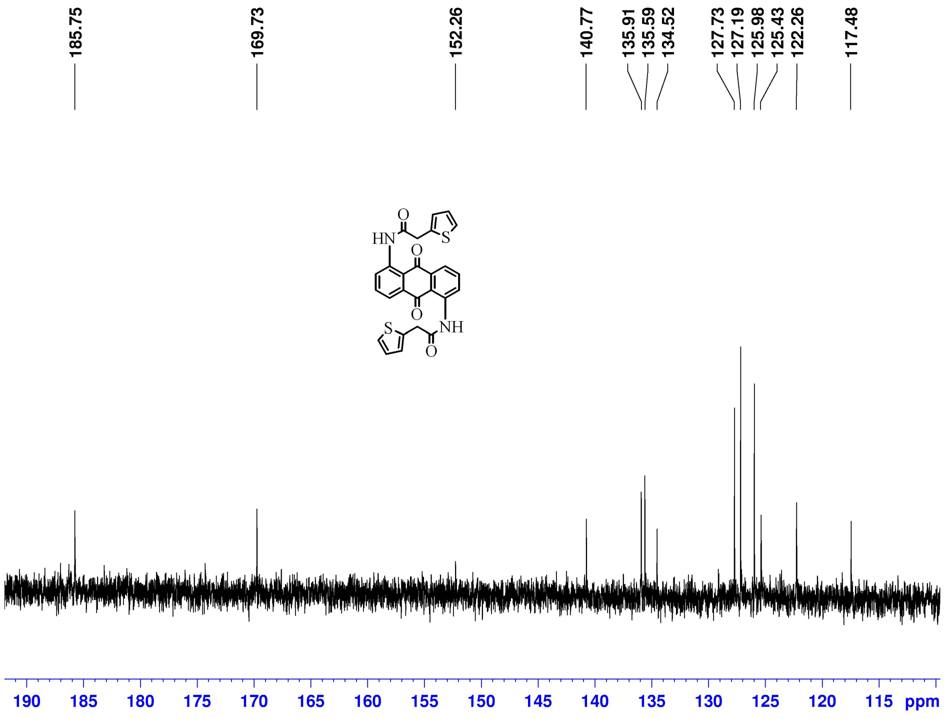

Figure S8. ^{13}C NMR spectrum of **3b** (DMSO-d_6, 125 MHz, 298K).

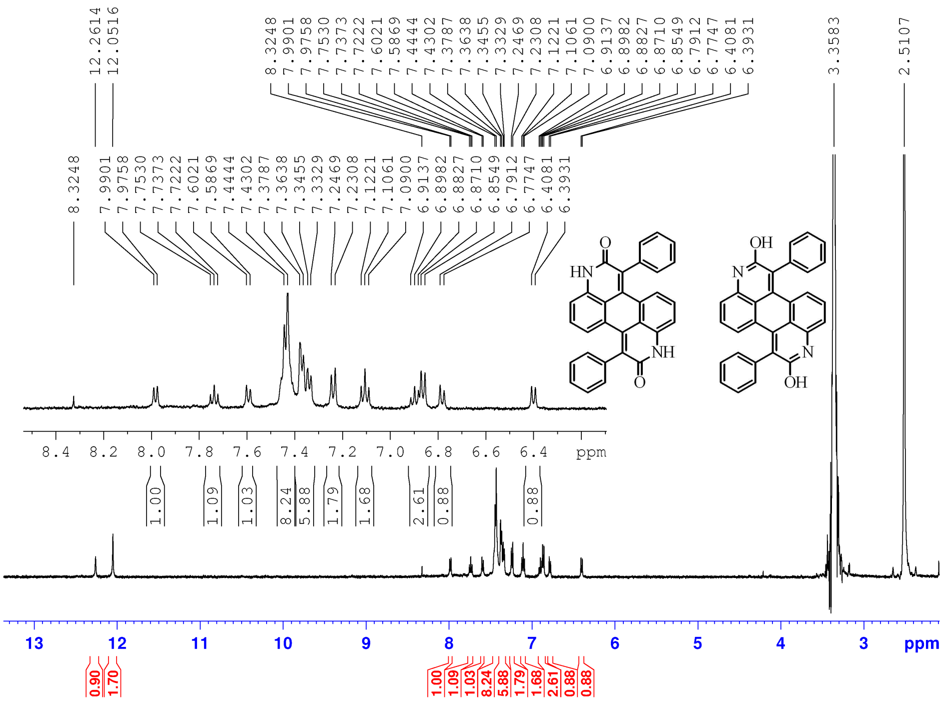

Figure S9. ^{1}H NMR spectrum of **4a** (DMSO-d$_6$, 500 MHz, 298K).

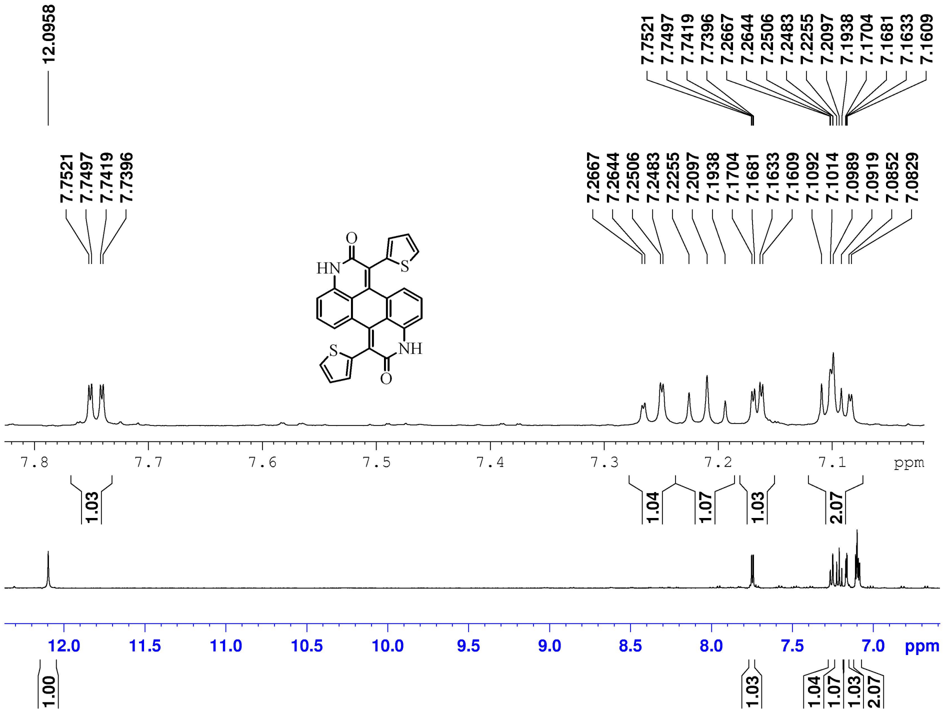

Figure S10. ^{1}H NMR spectrum of **4b** (DMSO-d$_6$, 500 MHz, 298K).

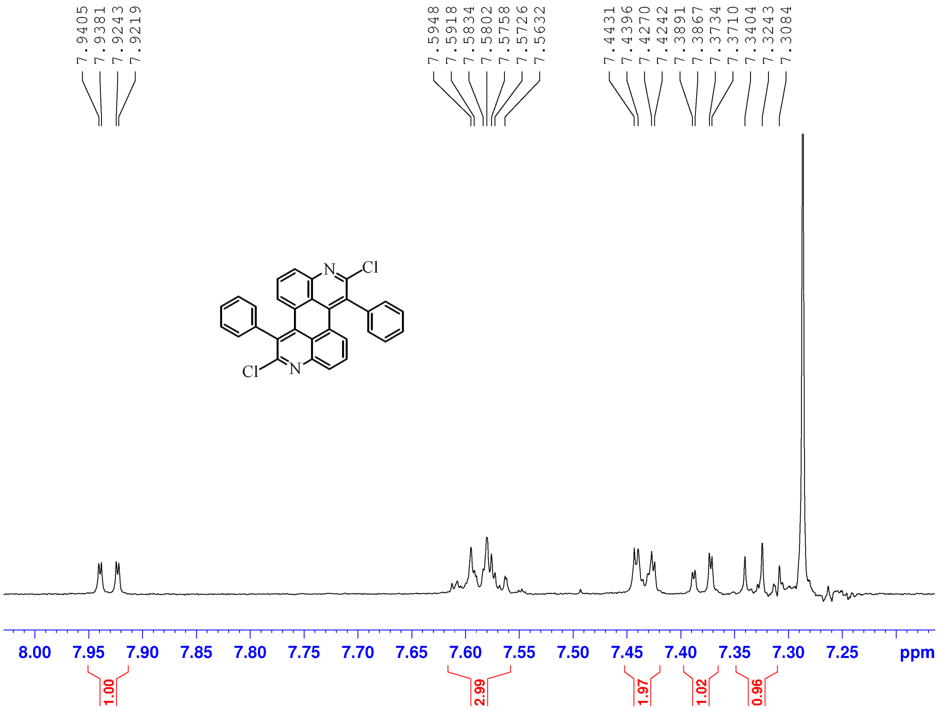

Figure S11. ^{1}H NMR spectrum of **5a** ($CDCl_3$, 500 MHz, 298K).

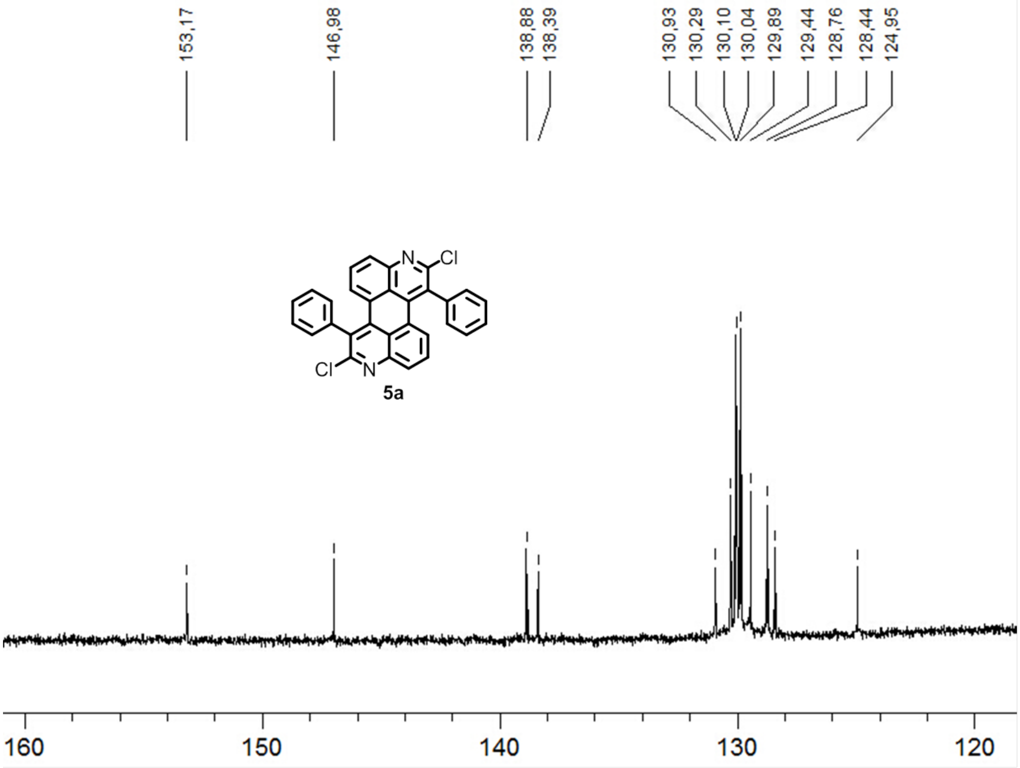

Figure S12. ^{13}C NMR spectrum of **5a** ($CDCl_3$, 125 MHz, 298K).

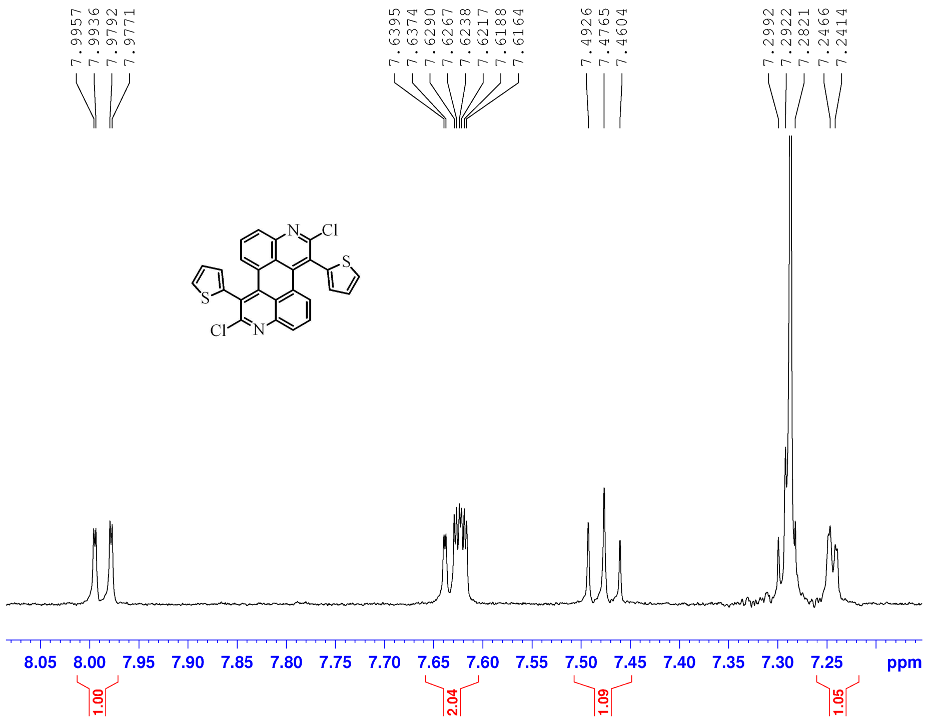

Figure S13. ^{1}H NMR spectrum of **5b** ($CDCl_3$, 500 MHz, 298K).

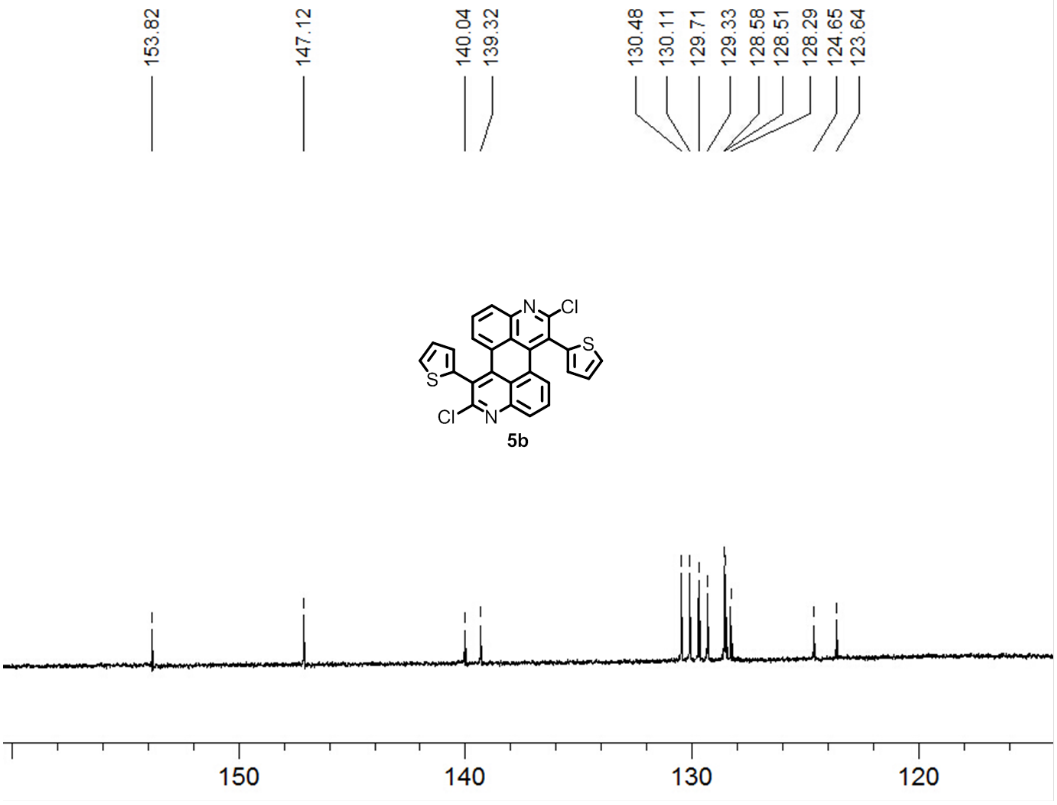

Figure S14. ^{13}C NMR spectrum of **5b** ($CDCl_3$, 125 MHz, 298K).

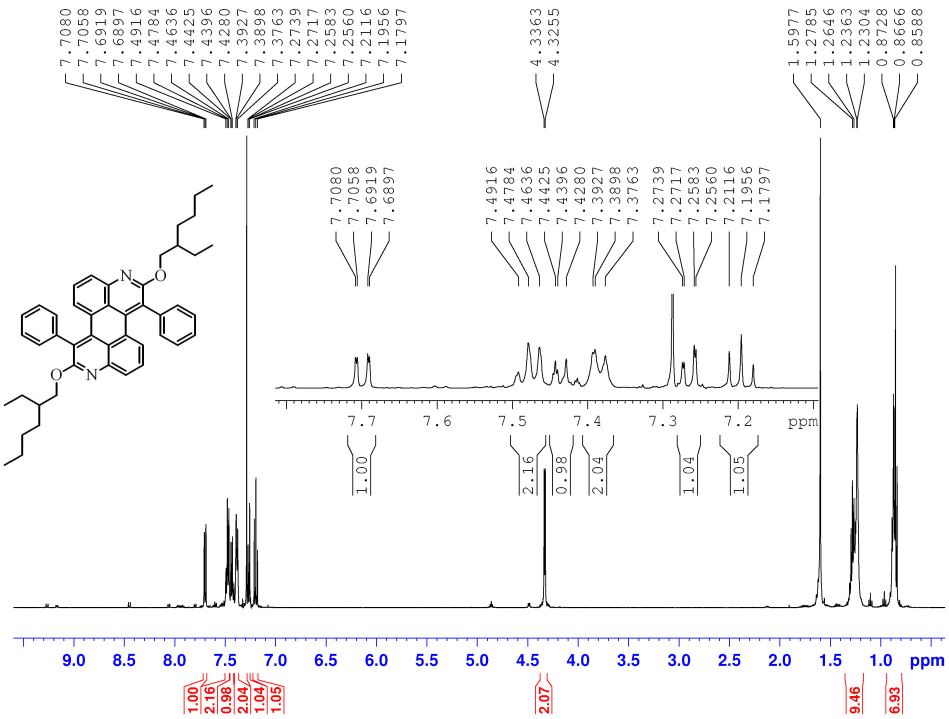

Figure S15. ^{1}H NMR spectrum of **6** ($CDCl_3$, 500 MHz, 298K).

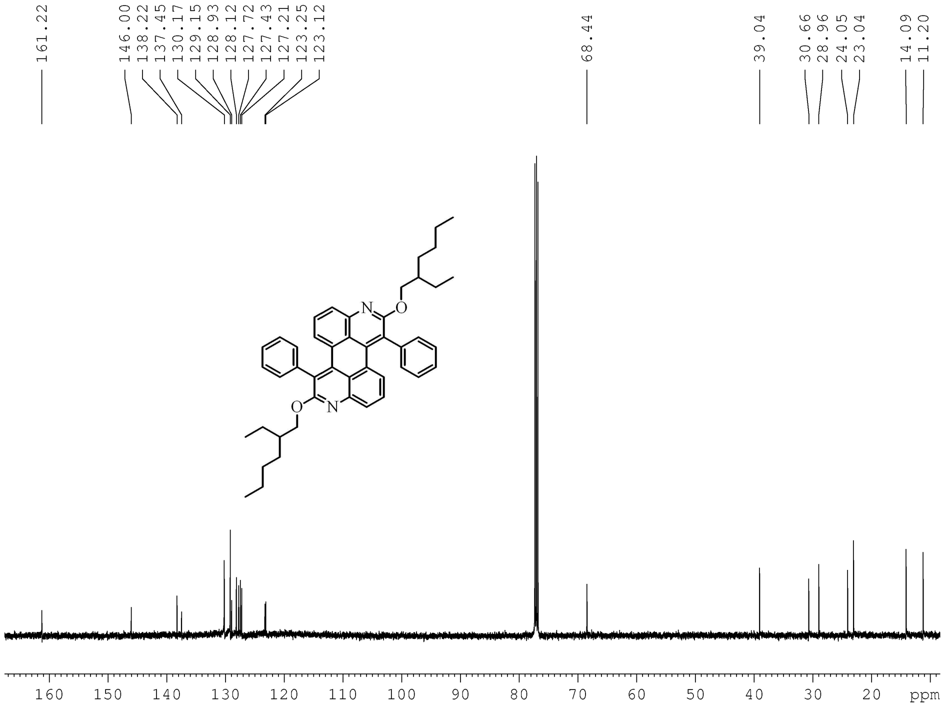

Figure S16. ^{13}C NMR spectrum of **6** ($CDCl_3$, 125 MHz, 298K).

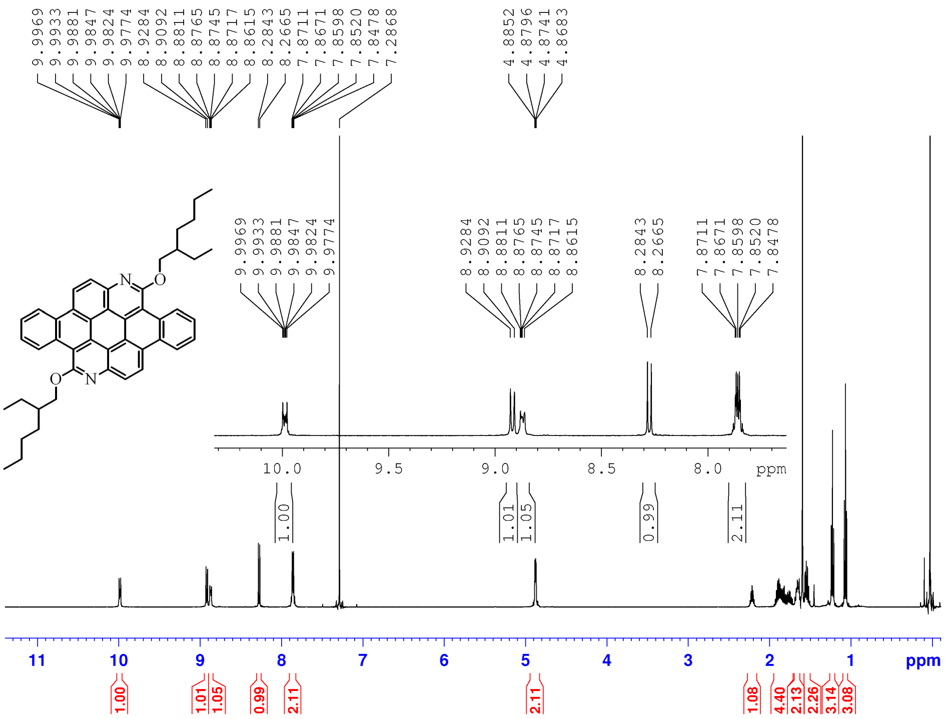

Figure S17. ^{1}H NMR spectrum of **7** ($CDCl_3$, 500 MHz, 298K).

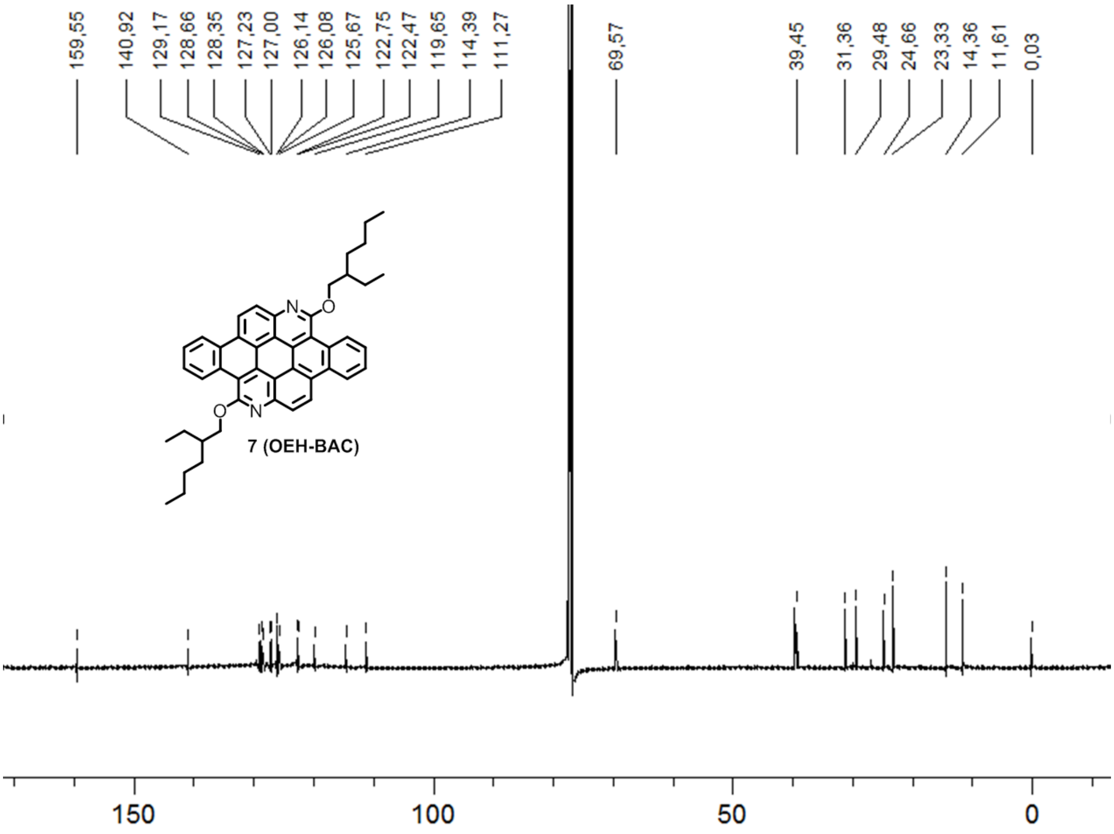

Figure S18. ^{13}C NMR spectrum of **7** ($CDCl_3$, 125 MHz, 298K).

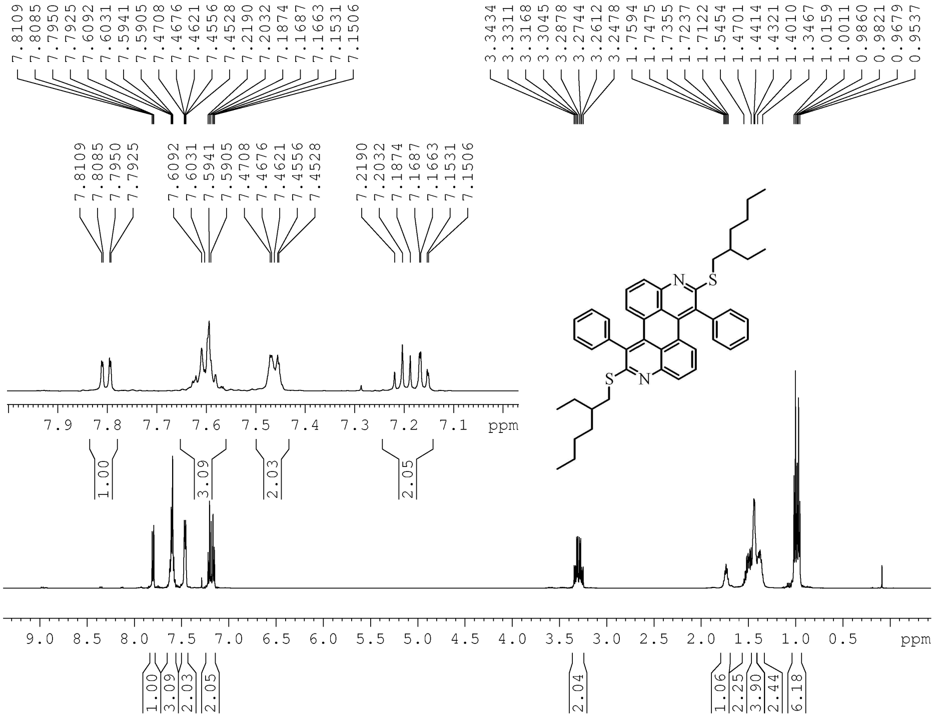

Figure S19. ^{1}H NMR spectrum of **8** ($CDCl_3$, 500 MHz, 298K).

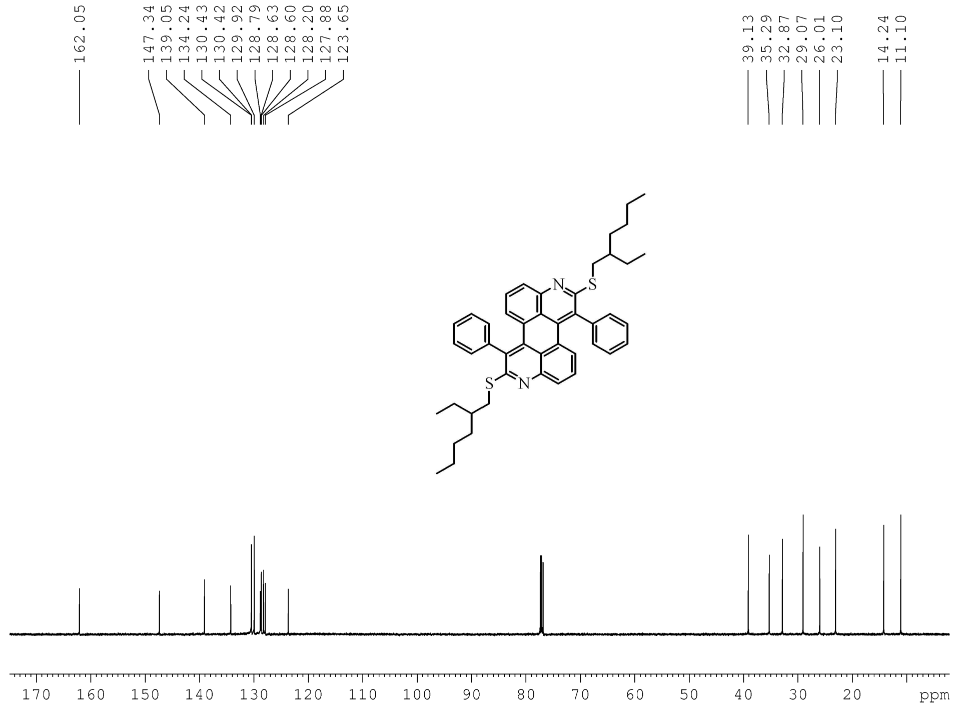

Figure S20. ^{13}C NMR spectrum of **8** ($CDCl_3$, 125 MHz, 298K).

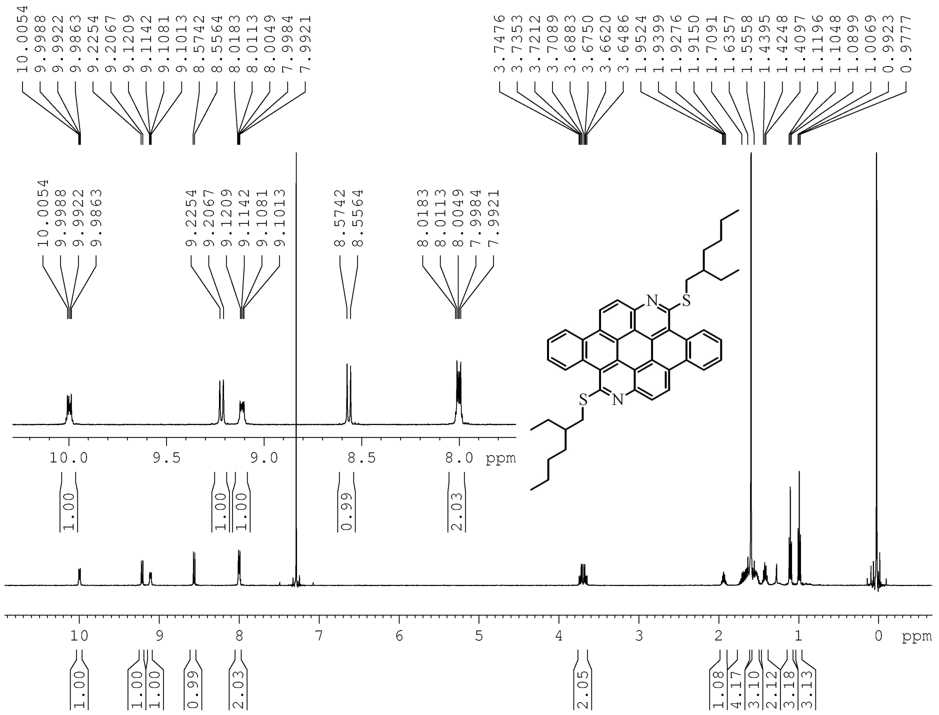

Figure S21. ^{1}H NMR spectrum of **9** ($CDCl_3$, 500 MHz, 298K).

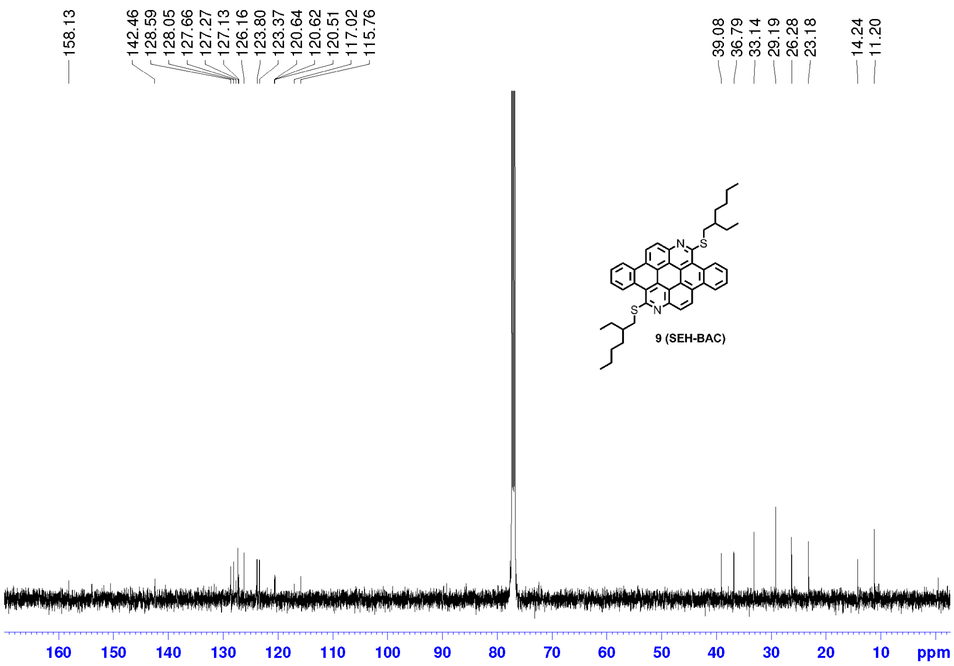

Figure S22. ^{13}C NMR spectrum of **9** ($CDCl_3$, 125 MHz, 323K).

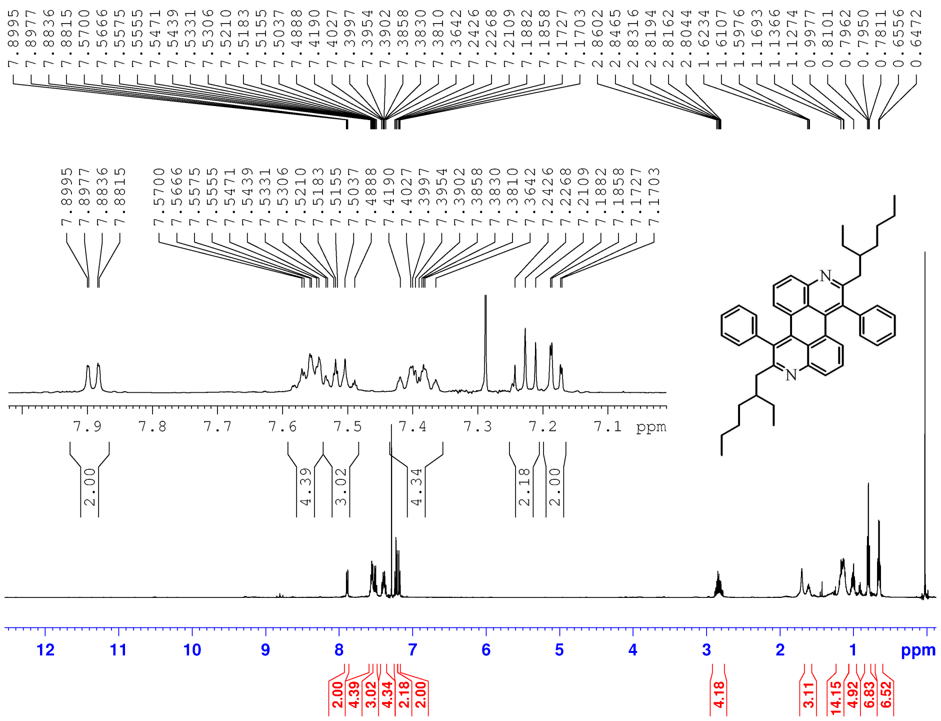

Figure S23. ¹H NMR spectrum of **10** ($CDCl_3$, 500 MHz, 298K).

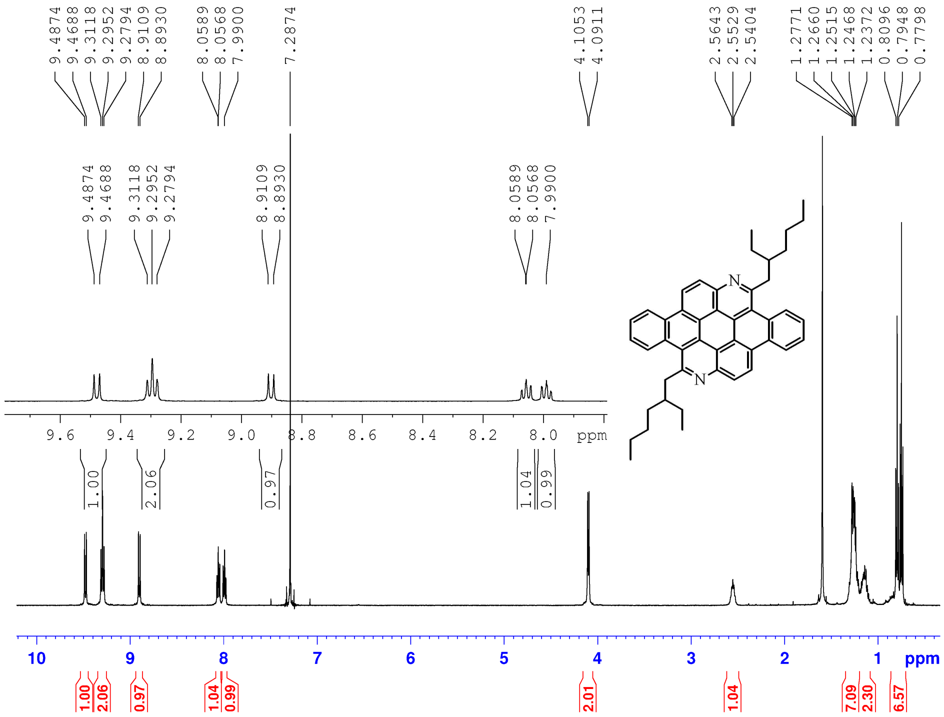

Figure S24. ¹H NMR spectrum of **11** ($CDCl_3$, 500 MHz, 298K).

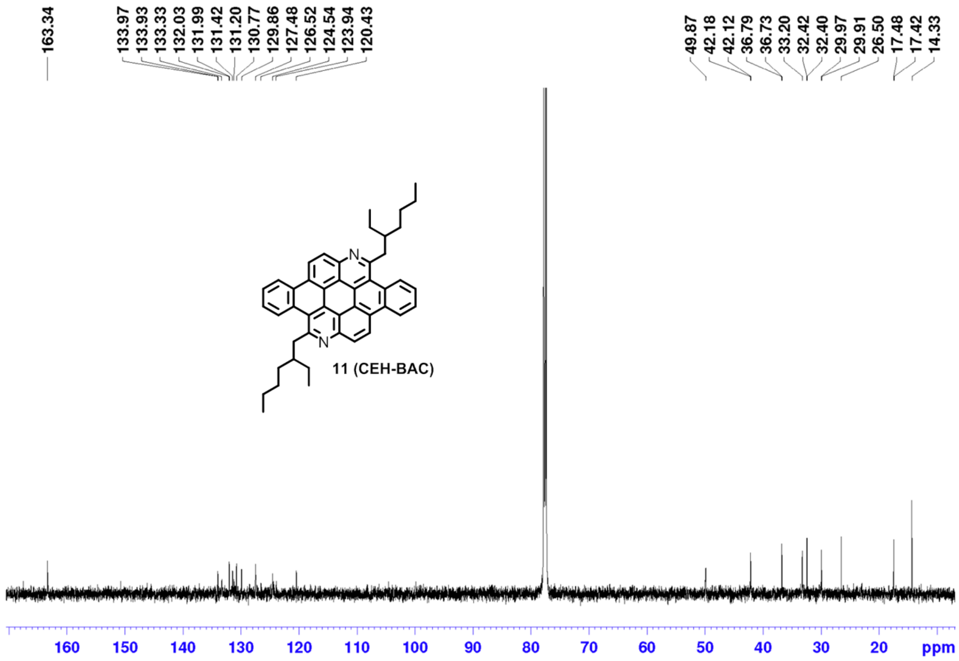

Figure S25. ^{13}C NMR spectrum of **11** ($CDCl_3$, 125 MHz, 323K).

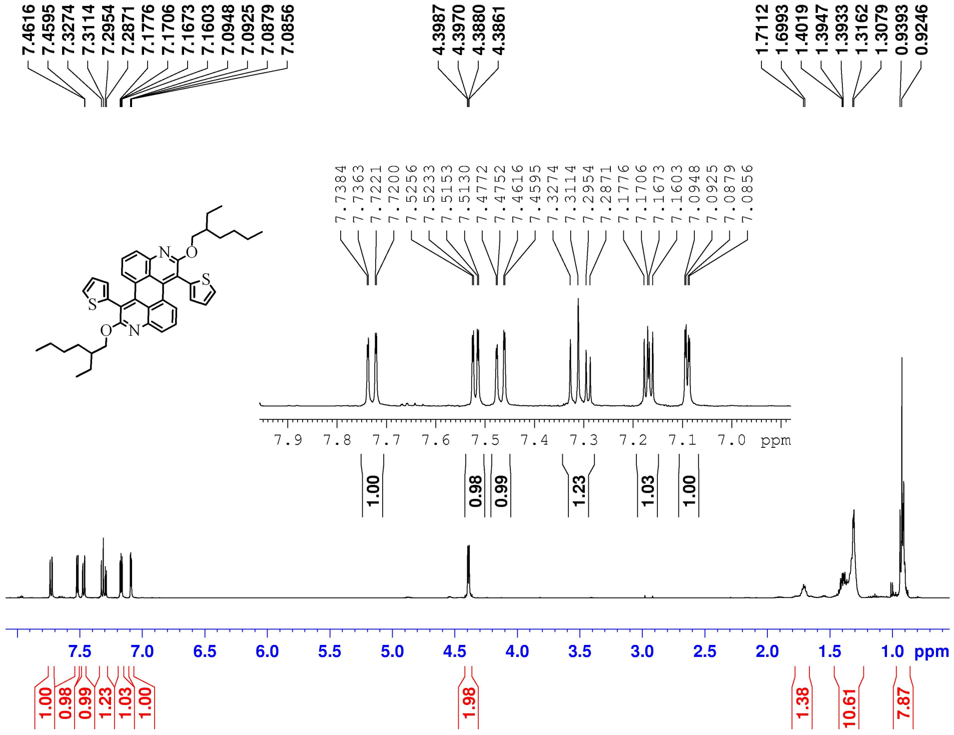

Figure S26. ^{1}H NMR spectrum of **12** ($CDCl_3$, 500 MHz, 298K).

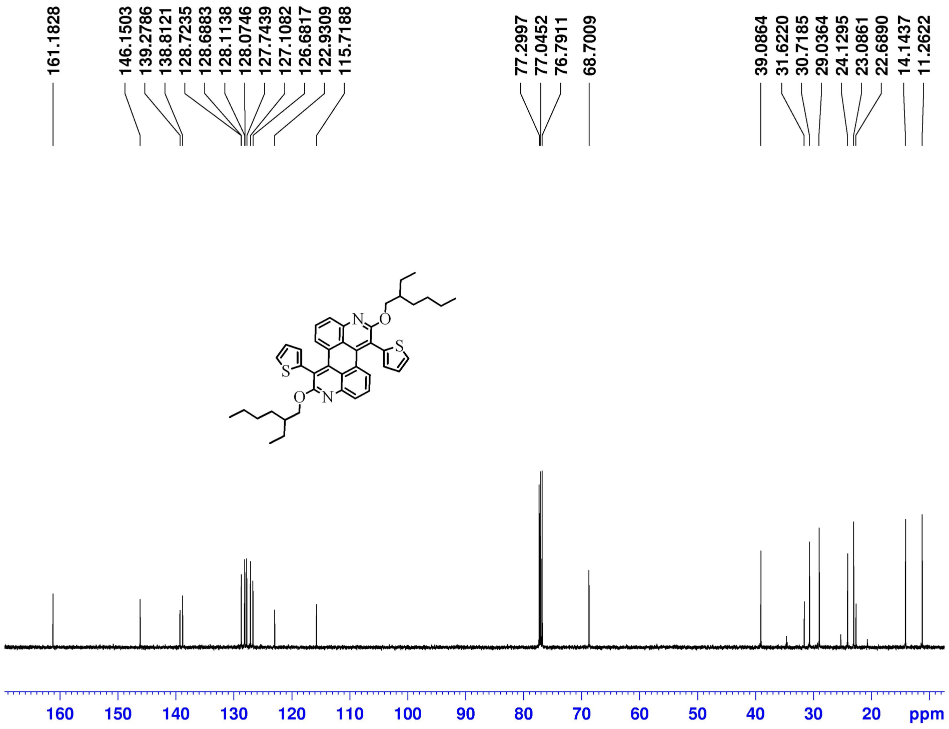

Figure S27. ^{13}C NMR spectrum of **12** ($CDCl_3$, 125 MHz, 298K).

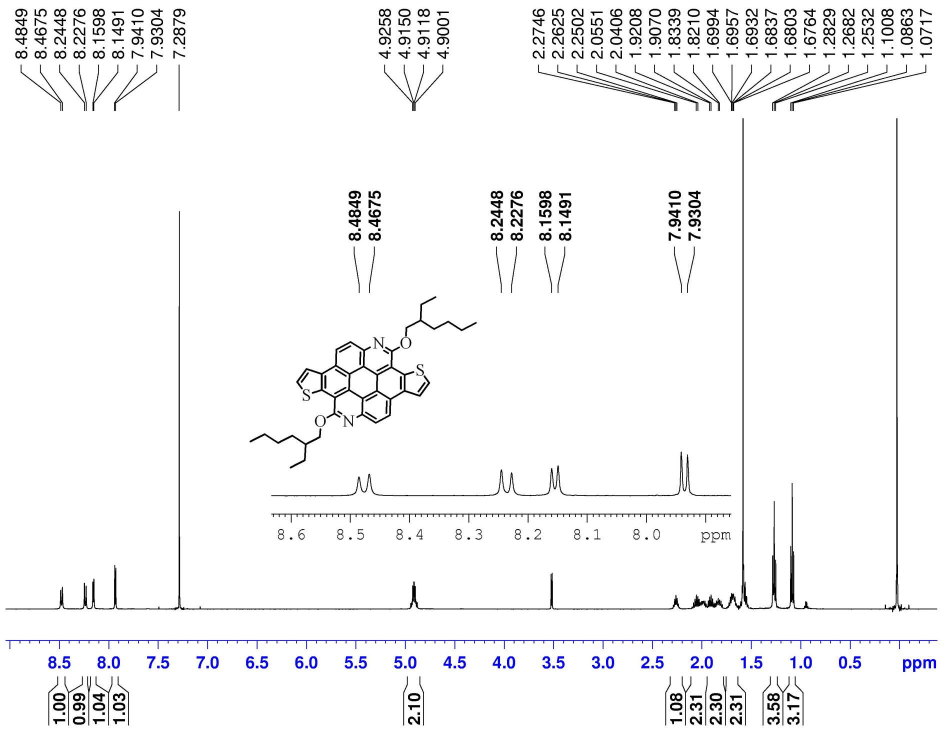

Figure S28. ^{1}H NMR spectrum of **13** ($CDCl_3$, 500 MHz, 298K).

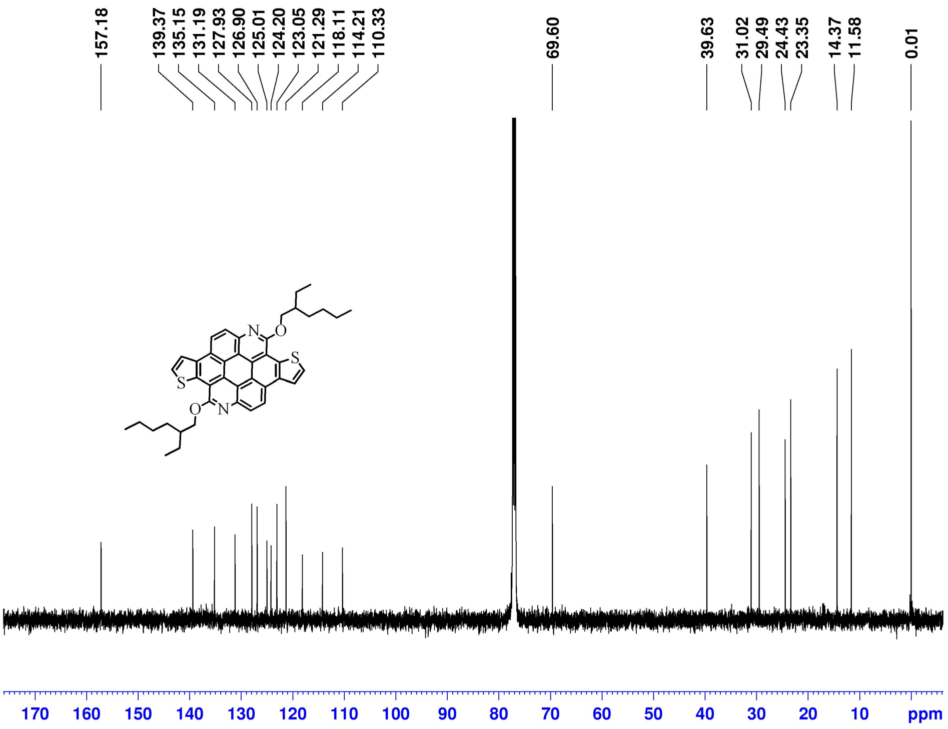

Figure S29. ^{13}C NMR spectrum of **13** ($CDCl_3$, 125 MHz, 298K).

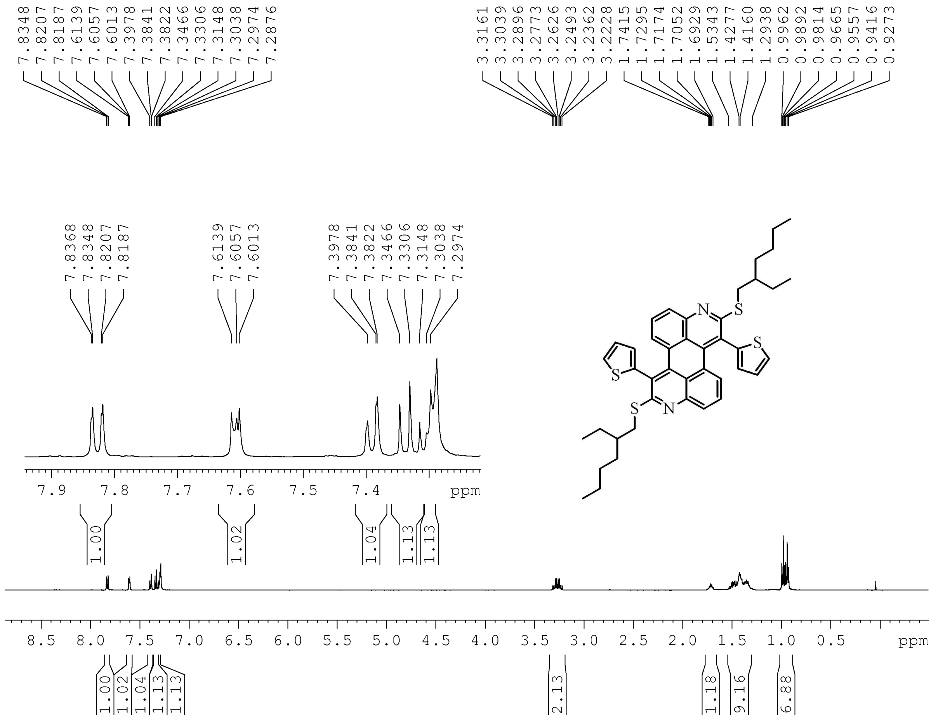

Figure S30. ^{1}H NMR spectrum of **14a** ($CDCl_3$, 500 MHz, 298K).

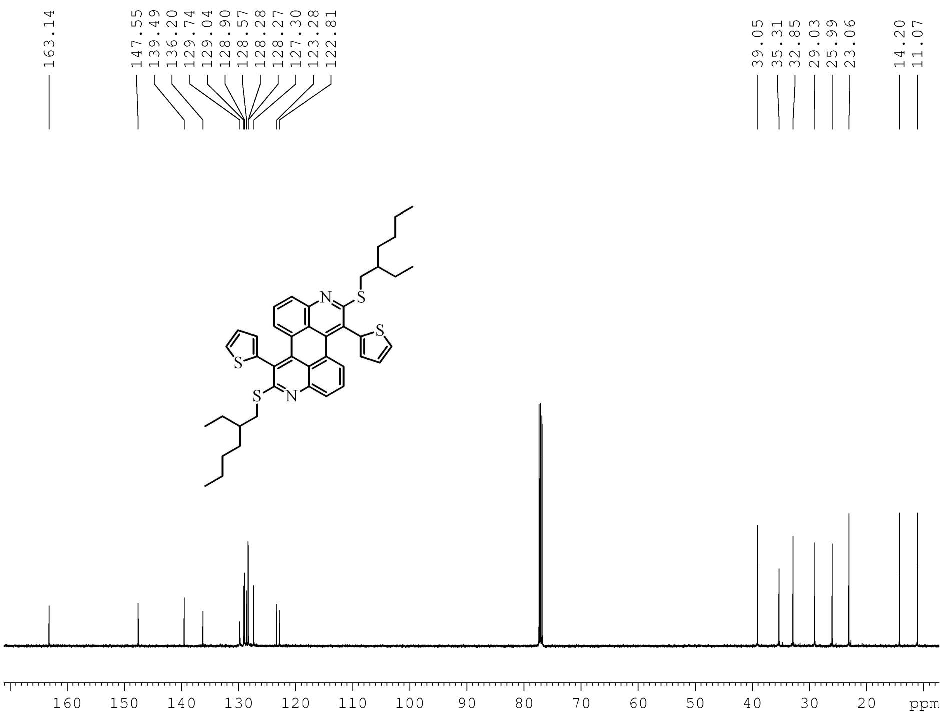

Figure S31. ^{13}C NMR spectrum of **14a** ($CDCl_3$, 125 MHz, 298K).

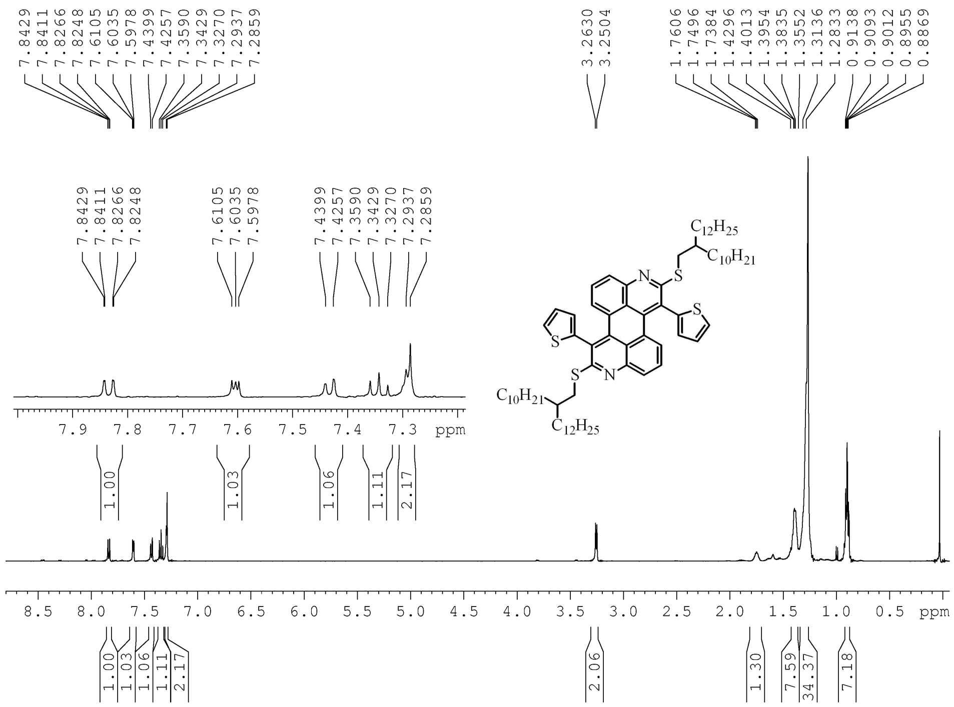

Figure S32. ^{1}H NMR spectrum of **14b** ($CDCl_3$, 500 MHz, 298K).

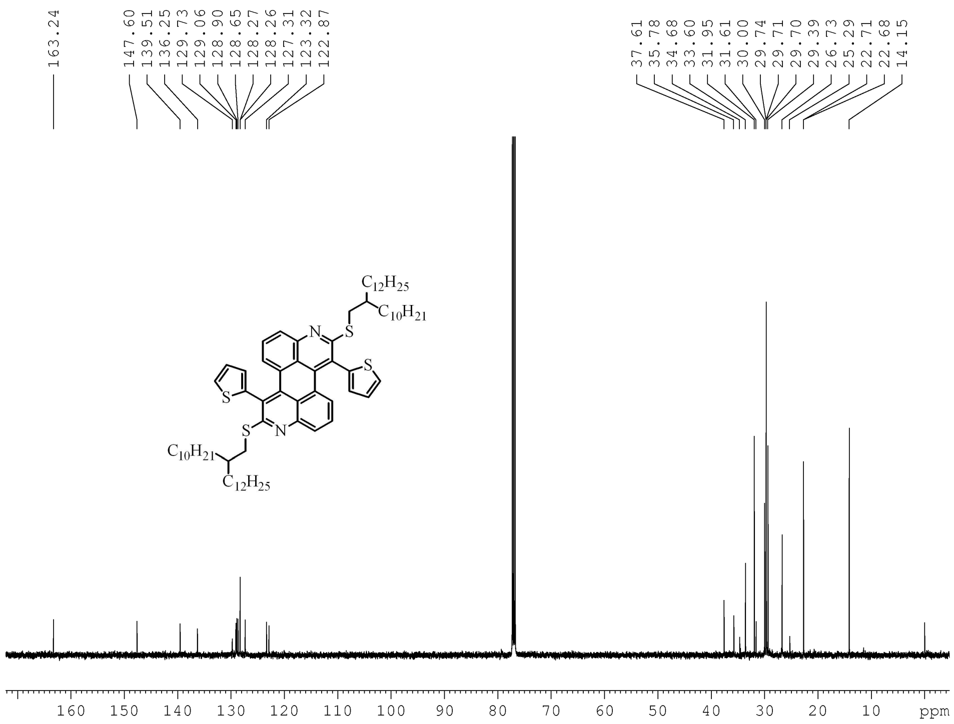

Figure S33. ^{13}C NMR spectrum of **14b** ($CDCl_3$, 125 MHz, 298K).

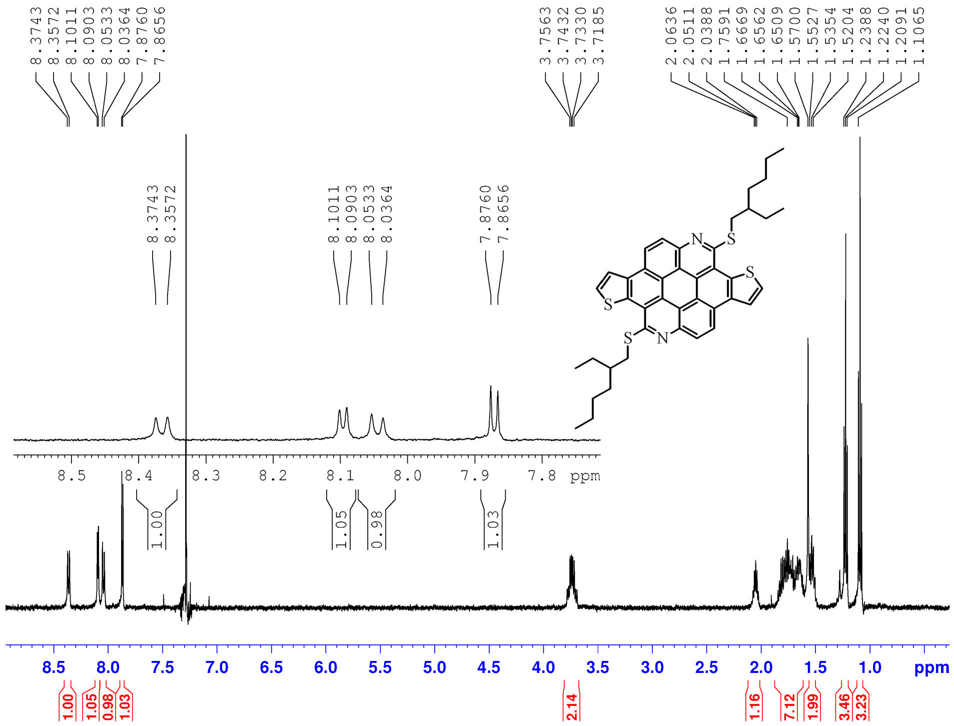

Figure S34. ^{1}H NMR spectrum of **15a** ($CDCl_3$, 500 MHz, 298K).

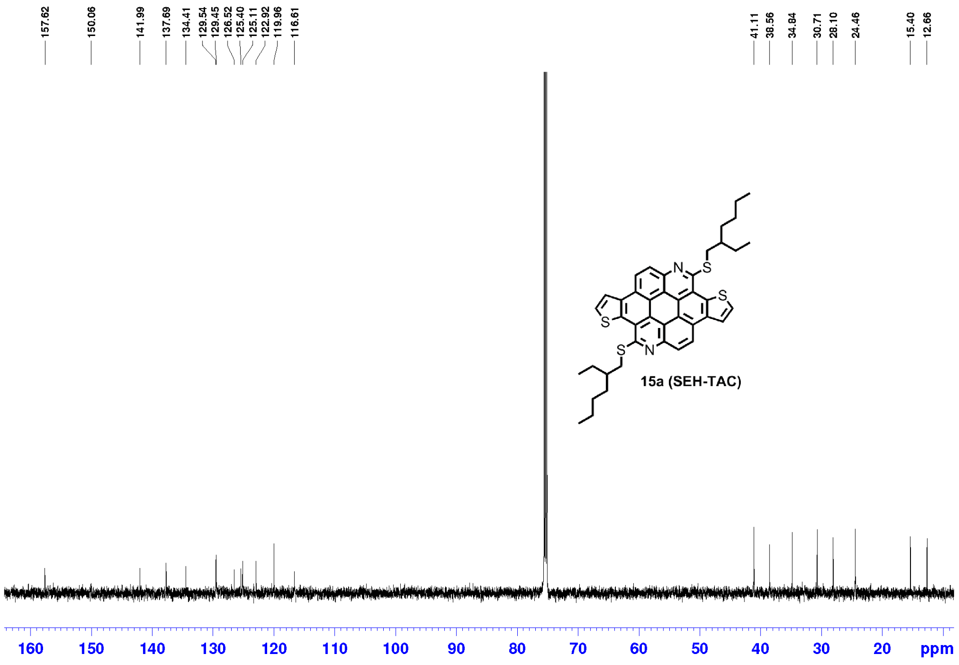

Figure S35. ^{13}C NMR spectrum of **15a** ($CDCl_3$, 125 MHz, 323K).

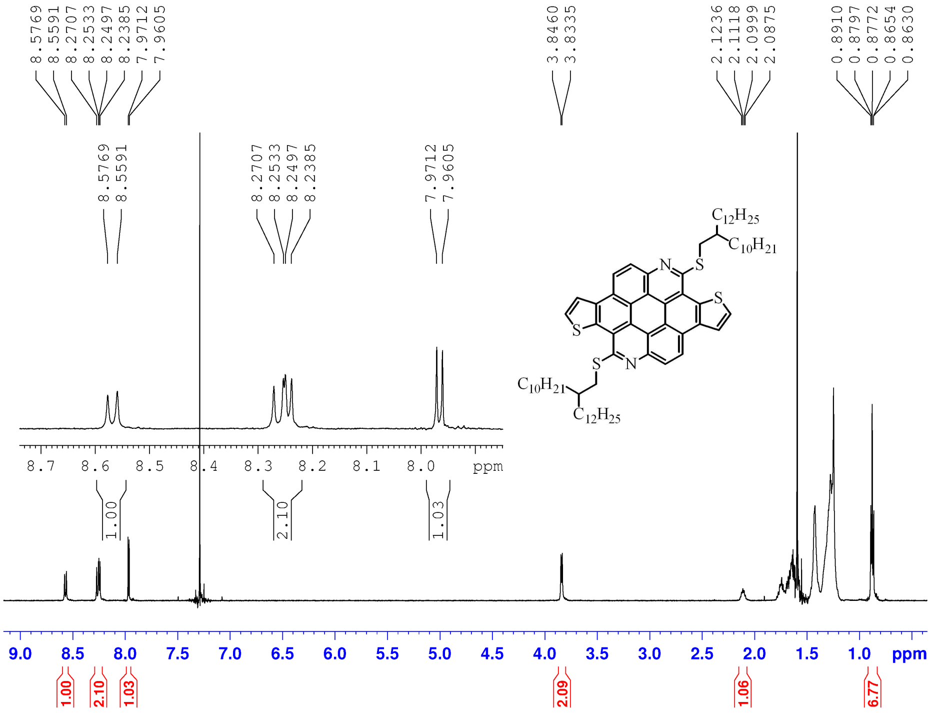

Figure S36. ^{1}H NMR spectrum of **15b** ($CDCl_3$, 500 MHz, 298K).

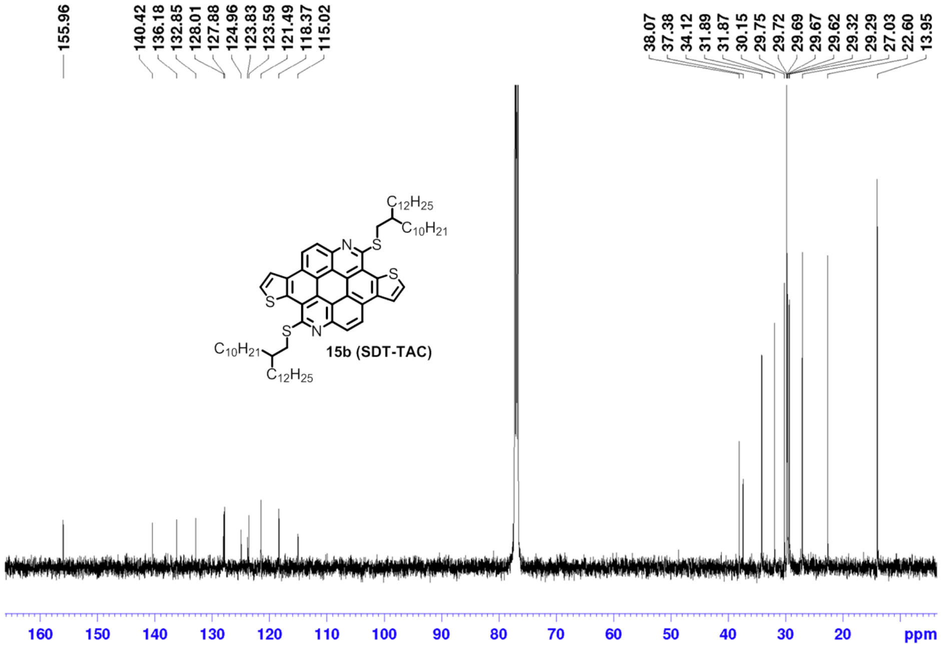

Figure S37. ^{13}C NMR spectrum of **15b** ($CDCl_3$, 125 MHz, 323K).

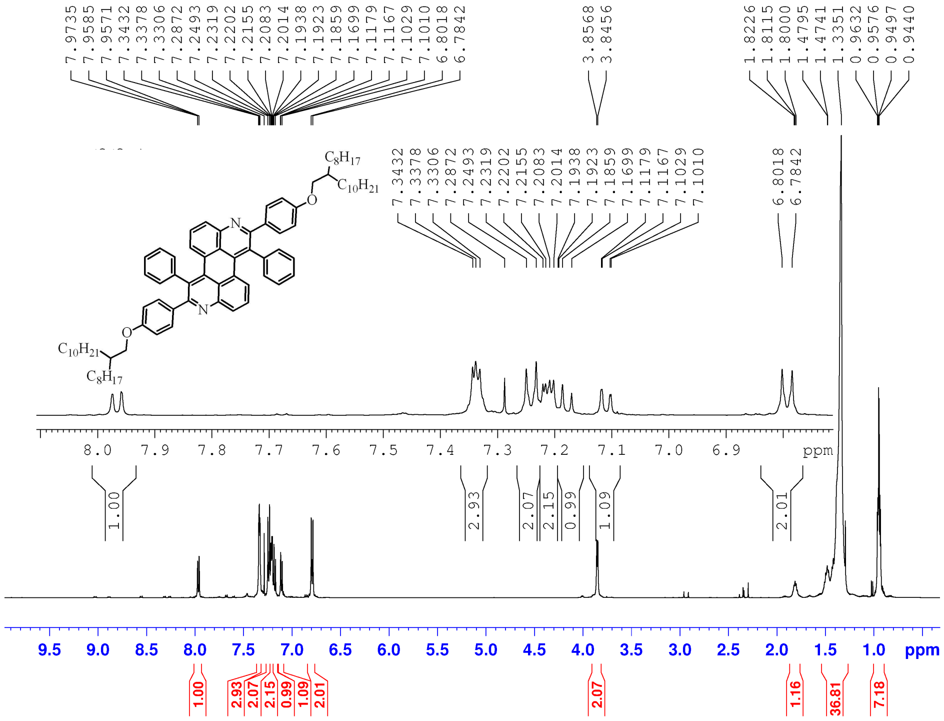

Figure S38. ^{1}H NMR spectrum of **16** ($CDCl_3$, 500 MHz, 298K).

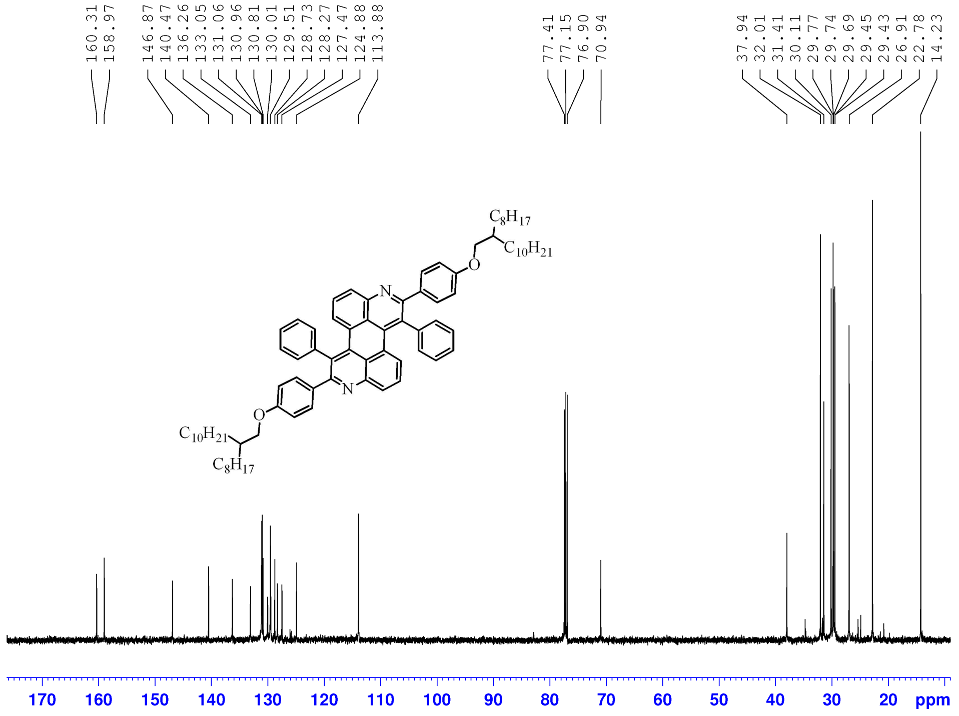

Figure S39. ^{13}C NMR spectrum of **16** ($CDCl_3$, 125 MHz, 298K).

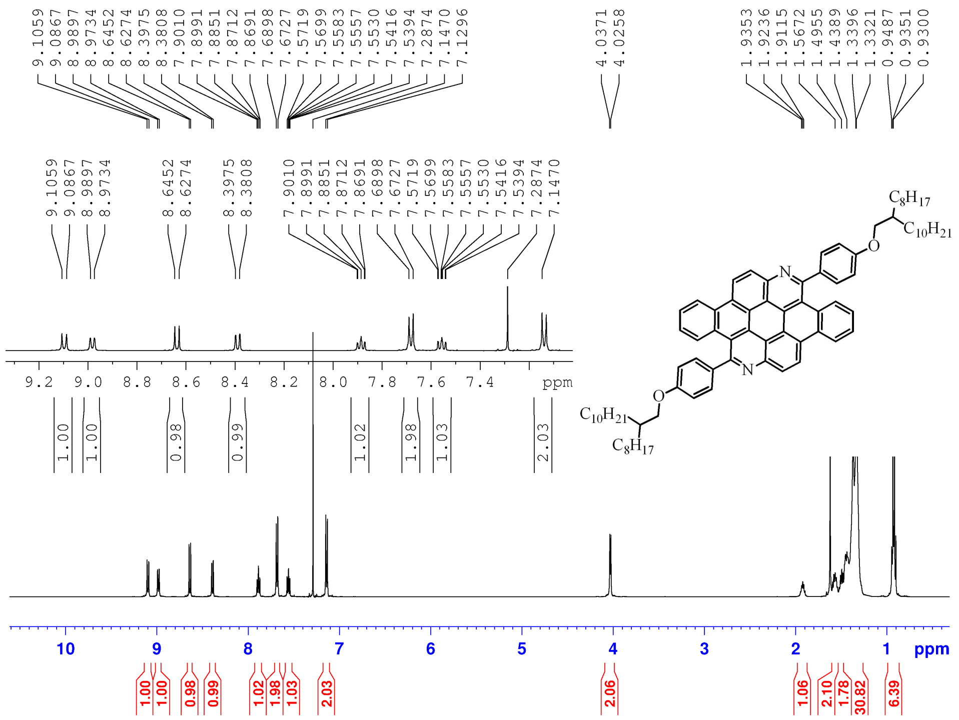

Figure S40. ^{1}H NMR spectrum of **17** ($CDCl_3$, 500 MHz, 298K).

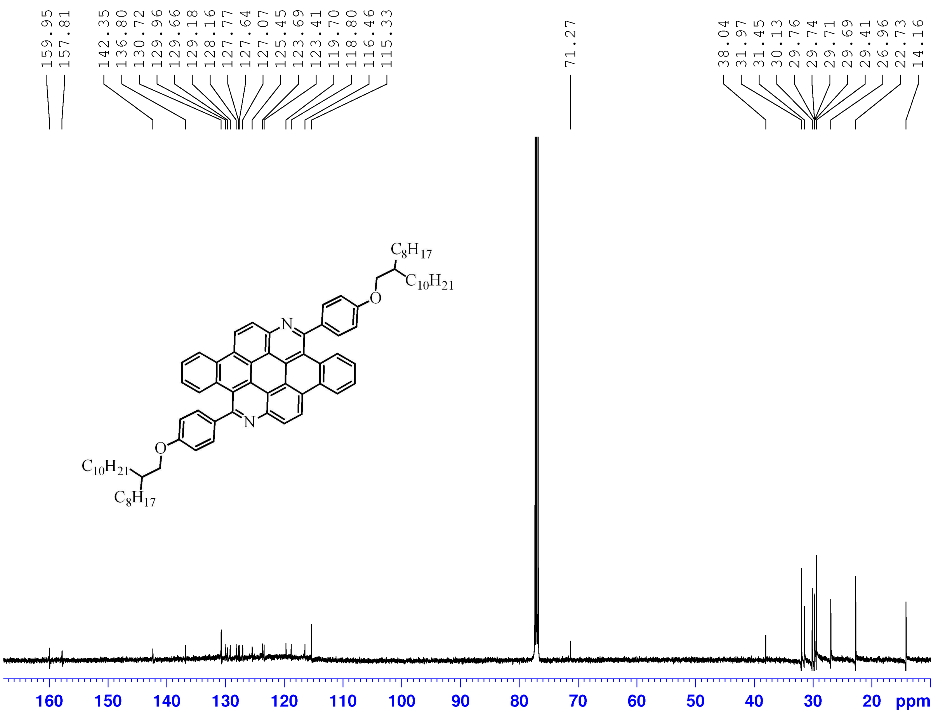

Figure S41. ^{13}C NMR spectrum of **17** ($CDCl_3$, 125 MHz, 298K).
